# Advances in supporting development in autistic children and youth

**DOI:** 10.1136/bmj-2025-086562

**Published:** 2026-06-10

**Authors:** Melanie Penner, Lonnie Zwaigenbaum, Nicholas Piroddi, Saebom Park, Ripudaman Singh Minhas, Deepa Singal

**Affiliations:** 1 Holland Bloorview Kids Rehabilitation Hospital, Toronto, Ontario, Canada, M4G 1R8; 2 Department of Paediatrics, University of Toronto, Toronto, Ontario, Canada, M5G 1X8; 3 Autism Research Centre, Glenrose Rehabilitation Hospital, Edmonton, Alberta, Canada, T5G 0B7; 4 Department of Pediatrics, University of Alberta, 116 St and 85 Ave, Edmonton, Alberta, Canada, T6G 2R3; 5 Institute of Medical Science, University of Toronto, Medical Sciences Building, Toronto, Ontario, Canada, M5S 3K3; 6 Department of Pediatrics, St. Michael’s Hospital, Unity Health Toronto, 30 Bond St, Toronto, Ontario, Canada, M5B 1W8; 7 Autism Alliance of Canada, PO Box 43081, RPO Sheppard Centre, North York, Ontario, Canada, M2N 6N1

## Abstract

Autism is a neurodevelopmental condition with varied trajectories through the lifespan, leading to individualized patterns of strengths and challenges. Longitudinal autism cohort studies show the importance of developmental and adaptive skills starting in the early years, followed by emerging co-occurring conditions, and opportunities for autonomy and community participation when approaching adulthood. Studies of interventions to support developmental outcomes in autistic children have shown benefits; however, adverse events from therapies and outcomes prioritized by autistic people must be incorporated. Programs for autistic children and youth are making some progress by including members of diverse communities, developing and adapting interventions to meet their needs. Most importantly, autistic people have highlighted the many contributors to a ‘good life’, prominent among which are acceptance and meaningful inclusion. This review summarizes the latest evidence about developmental trajectories and outcomes among autistic children and youth, and how this translates into clinical practice and policy.

## Introduction

**Nomenclature**

Aligned with feedback from many autistic individuals and advocacy organizations, while acknowledging that preferences vary, this review adopts identity-first language (such as “autistic person”).

In this narrative review, we aim to describe the state of the science as it applies to supporting best possible outcomes for autistic children and youths. Autism is defined by challenges with social communication and the presence of restricted or repetitive partners of behavior or interest.^1^ As more autistic children and youths access healthcare and related services, there is a growing need for evidence informed approaches that recognize their strengths, support diverse developmental pathways, and promote meaningful outcomes.

Understanding the factors that shape the diverse trajectories experienced by autistic children and adolescents can help clinicians and policymakers create more responsive and inclusive support.^2^ This review synthesizes current evidence on developmental trajectories and outcomes among autistic children and adolescents and identifies key gaps and priorities to inform clinical practice, future research, and policy. We aim to combine this synthesis with key concepts of intersectionality and affirmation of neurodivergence, or neurodevelopmental profiles that are outside of what is considered “typical,”^3^ producing a holistic review of the state of autism science and future directions.

This review is structured around the following three guiding questions:

1. What factors influence developmental trajectories and outcomes among autistic children and adolescents?
2. What therapy based interventions positively influence developmental outcomes among autistic children and adolescents?
3. How do contextual factors, such as sex, gender, culture, and race/ethnicity influence autistic development and outcomes?

## Epidemiology

Recent studies estimate that approximately 1-2% of children worldwide are autistic.^4^ ^5^ ^6^ A review of autism epidemiological studies published since 2012 reported a median prevalence of 1%.^6^ Studies have also shown a continued increase in the reported prevalence of autism. In the United States, 2022 surveillance data from the Autism and Developmental Disabilities Monitoring (ADDM) Network reported that, among 8 year old children, 1 in 31 were autistic, an increase from 1 in 36 in 2020.^4^ These trends are widely attributed to greater awareness, expanded diagnostic criteria, and improved access to assessment and services rather than a definitive increase in incidence.^4^ ^5^ ^6^

Subgroup patterns also show considerable variability in prevalence. The median male-to-female ratio across studies is 4.2 to 1, although emerging evidence indicates that autistic people assigned female at birth may remain under-identified due to differences in behavioral presentation and diagnostic biases.^6^ Recent ADDM reports indicate equal or higher prevalence among Black, Hispanic, and Asian/Pacific Islander children compared with White children, reflecting shifts in diagnostic equity for historically under-identified groups.^5^ Among those in the ADDM sample with information about cognitive ability, nearly 40% were classified as having an intellectual disability.^5^ Asian/Pacific Islander, Black, and Hispanic autistic children were more likely to have co-occurring intellectual disability compared with White or multiracial autistic children, which may reflect disparities in access to diagnostic services for children without intellectual disability.^5^

## Methods

### Author positionality

The authors have varied professional backgrounds and lived experiences, and include clinician-scientists in developmental pediatrics, graduate trainees, and health services and policy researchers. We represent a range of racial and gender identities and include both neurotypical and autistic perspectives. We acknowledge that our positionality may have influenced how we interspersed and synthesized the evidence in this review. Throughout, we have aimed to center strengths based, respectful, and inclusive language, while recognizing the diversity of perspectives within the autism community.

Our review is informed by biomedical and social models of disability, which each offer important perspectives to inform clinical practice and policy for autistic people. The biomedical model focuses on diagnosis, individual level challenges, and targeted interventions, whereas the social model emphasizes the role of external barriers, such as inaccessible environments and societal attitudes that limit participation of autistic people. For autistic individuals, many challenges arise from a mismatch between individual characteristics and the social and physical environment, rather than from the condition itself. Methodological advances in the field, including participatory research designs that amplify autistic voices, have contributed to seismic shifts in the conceptualization of “optimal” outcomes for autistic children and youth. While traditional models often prioritized independence as a primary goal, contemporary autism research increasingly acknowledges interdependence as part of our shared human experience. This evolving understanding supports a more holistic and personalized view of what constitutes a “good” autistic life.

### Literature search

We searched PubMed, Scopus, Web of Science, and Google Scholar using tailored keyword combinations. All search strategies extended until 31 May 2025 and are provided in Appendix 1.

*Longitudinal cohort studies—*We searched for systematic and scoping reviews on observational longitudinal studies involving autistic children and youth conducted between 1 January 2014 and 31 May 2025. Reviews were included if they reported developmental outcomes measured over two or more time points in childhood or adolescence. We did not restrict by developmental domain, aiming to capture breadth of outcomes reported in the literature. Reviews focused primarily on adults were excluded. Where reviews included mixed age samples (such as children and adults), findings relevant to children, youth, and young adults (<24 years old) were extracted.

*Intervention studies—*We searched for systematic reviews on intervention studies involving autistic children and youth published between 1 January 2019 and 31 May 2025. A narrower time frame was applied for inclusion of intervention studies due to the volume of reviews, and to anchor the time with the publication of a landmark review.^7^ We included reviews of non-pharmacological interventions that reported effect sizes and that focused primarily on autism related outcomes, including core features, related developmental domains, and mental health. Reviews centered on biomedical interventions, pharmacological, or co-occurring medical conditions (such as sleep disorders) were excluded. Studies were stratified by age (early childhood, school age, adolescence) to support interpretation of outcomes across age groups.

Additional searches focused on identifying relevant reviews in specific topic areas (see Appendix 1). We searched for systematic and scoping reviews that focused on community based participatory research for autistic children/youth. We searched systematic and scoping reviews on the intersection between sex, gender, and autism to help contextualize findings of the longitudinal cohort studies and intervention studies. We searched for systematic and scoping reviews of intervention studies involving autistic children and youth from marginalized groups, including Black, Indigenous, and other People of Color (BIPOC), racialized, minoritized, immigrant, or refugee communities. Additionally, we searched for interventions that were developed for use in low and middle income countries (LMICs).

## Developmental outcomes and trajectories for autistic children and youth

Reviews of longitudinal cohort studies provide a way to understand developmental outcomes and trajectories, or patterns over time. Such studies can be used to give parents of young children a general idea of what to expect in the coming years. Trajectory methods allow for the identification of multiple subgroups characterized by differing levels of an outcome that change differently over time. Non-trajectory methods provide insight into the general trend using the average of an earlier measure and the average of a later outcome for all individuals in a study sample.

### Outcome domains

Across the included reviews, a wide range of outcomes and measures were used to examine trajectories among autistic children and adolescents (table 1).^8^ ^9^ ^10^ ^11^ ^12^ ^13^ Common outcomes included adaptive behavior functioning (a measure of varied developmental skills), social functioning, autism features, externalizing behaviors, and executive function. Numerous tools and measures were used to assess outcomes, and the decision to report composite scores or subscale scores for a given measure (such as Child Behavior Checklist) differed between studies. The variability in outcome domain and measurement approaches highlights the lack of standardization in the field and complicates cross-study comparisons.

**Table 1:** Summary of reviews on longitudinal studies of autism

Study, review type	Reported sample characteristics	Reported methodological characteristics	Outcome (measures)	Key findings
Gentles (2024)^8^ Scoping review of trajectory studies	No of studies 103 Sample size 17-8653 (sample size for autistic individuals 10-6975) Age range 6-312 months	Sampling methods: community based (36.9%); clinical (36.9%); population based (9.7%); NR (16.5%) Design of included studies: 96 prospective; 7 retrospective analyses using cohort data	*Adaptive behavior functioning* (VABS-1, −2 composite and subdomains; Vineland Social Maturity Scale) *Social functioning* (CDER Soc; VASBS-1, −2 Soc) *Communication* (VABS-1, −2 Com; CDER Com) *Daily living skills* (VABS-1, −2 DLS; *Autism features* (ADOS CSS, CSS-SA, -RRB, raw totals, total algorithm score, individual items; PDDBI; AUTISM-C; SRS-1; SEQ; ABC; ADI-R *Restricted and repetitive behaviors* (ADOS RRB total score; ADI-R: RSM, IS; RBS-R) *Internalizing problems* (The Mood List, EAQ, W/RQC, CSI, CDI, SCL; ASEBA: Anxiety, Affective/ Depression, CBCL/ABCL; CBCL/ABCL: Anxious/Depressed, Withdrawn/Depressed; DBC: Depressive; ECI-4; SDQ: Emotional Difficulties) *Externalizing problems* (CSI Disruptive behavior problems; ABCL: Hyperactivity, irritability, social withdrawal; ECI-4; SDQ Conduct problems, hyperactivity/ inattention, peer relationship problems; CBCL/ABCL Attention problems; RBS-R Self-injurious behaviors; Proprietary measure) *Cognitive functioning* (MP; WPPSI; MSEL DQ, ELC, NVIQ, VIQ, VR; WISC, −3; WASI-2; WASI-2 FSIQ, PRI, VIQ; ABAS-2; BSID; VPT NVIQ, VIQ) *Language* (PPVT-3; EOWPVT; PLS-3, −4 AC, EC; CASL; WJ-IIII-ACH-LWI; EVT-1; BPVS-2; VABS-2 EL, RL; MSEL EL, RL; Proprietary measure)	Studies were inconsistent in outcome assessment (number and timing) among participants. Social functioning, communication, daily living skills had good age coverage; cognitive functioning and language had good coverage <10 years. Inconsistency in age-referenced results between studies, with around 20% of studies failing to report trajectories in terms of age – results from these studies could not be compared with age-referenced results.
Adams (2023)^9^ Systematic review and meta-analysis of challenging behaviors	No of studies 56 Sample size 13-1603 Age range 6 months to 66 years (6 studies focused predominantly on adults with mean age at time 1 21.7-37.8 years (range 10-66) and follow-up period 1.5-8.5 years Average proportion of males 82.1% (4 studies NR) Average proportion of participants with ID 42.8% (8 studies excluded those with ID; in 3 studies, all had ID)	Time between baseline and final time point 9 months to 16 years Sampling methods cohorts (n=39); clinic/services (n=10); schools (n=4); NR (n=3)	*Challenging behaviors* (ABC Irritability, Agitation and Crying, Item 52, TBP, Total; Self-Injury Absent/Mild/Moderate/Severe Rating; SIB-R MBP, EXT, BPS, Total; Parent report during interview; CBCL Total, EXT, Aggression; CSI; Disruptive Behavior Scale; Peer nominations of disobedience; DBC TBP, MIS; NVBRF; TRF/CTRF EXT Problems, Total; SDQ Total Difficulties; CBQ SIB present/ absent; ECI-4 EXT; Conners Aggression; C21st Health Check Problem Behaviors Score; PCQ; Study specific measures; Non-standardized measures	Among studies that reported the change in challenging behavior over time, most studies noted a decrease; some found no change, some studies had mixed findings; 2/33 studies found an increase (note that it is unclear whether a decrease in scores of challenging behavior represents decrease in the number, frequency, or intensity of such behavior) 95% of included studies used standardized measures Broad measures of challenging behavior were the most commonly used (eg, CBCL); limited reports using subscale measures reflecting more specific behaviors (eg, self injury)
Tafolla and Lord (2024)^10^ Systematic review on mental health	No of studies 13 Sample size 65-6091 Age range 1.3-30.08 years (1 study captured outcomes from late childhood through to adulthood, with mean age in adulthood 25.73 years (range 23.17-30.08) Proportion of males 70-90%	Follow-up period 4-23 years *Sampling methods* community-based; clinical; population-based (proportions NR) Of 10/13 studies assessing symptoms of anxiety as primary outcome, 4 used trajectory analysis methods while 6 used non-trajectory analysis methods Of 6/13 studies assessing symptoms of depression as primary outcome, 3 used trajectory analysis methods while remaining half used non-trajectory analysis	*Anxiety* (CBCL Anxiety Problems, Affective Problems, RBS-R Ritualistic/Sameness; ADI-R IS; SCARED; SDQ Conduct Problems, Emotional Problems, Hyperactivity; ABCL Communication; DBC-Adult Anxiety, Depressive subtotals; DBC-Adult, -Parent; Anxiety Problems DSM-Oriented Scale; SEFI-R, Conners 3 *Depression* (CBCL Affective Problems, Depressive Problems; SDQ Conduct Problems, Depression, Emotional Problems, Hyperactivity; BDI-II; ABCL Depressive Problems; DBC-Adult Anxiety Disturbance, Depressive subtotals; SEFI-R; SMFQ; CIS-R	Higher adaptive behavior skills in childhood predicts lower symptoms of anxiety/ depression in adulthood Earlier symptoms of anxiety/ depression are strong predictors of greater anxiety/ depression symptoms later in development IS and RRBs in childhood predicts anxiety symptoms later in life Positive relationships with peers during school age appears to be protective against later depression symptoms in autistic adolescents Earlier maternal mental health could predict symptoms of depression in autistic adolescents and adults Inconsistent relationship between IQ and anxiety/ depression symptoms, as well as gender and anxiety/ depression symptoms
Rosello (2021)^11^ Systematic review on developmental outcomes	No of studies 28 Sample size 21-114 Age at intake 3-13.2 years Proportion of males over 80% in 17 studies; less than 70% in 3 studies	Follow-up period 1-11 years Design of included studies prospective	*Autism features* (ASSQ; ADOS module 3, module 4, module RRBI, ADOS-G, ADOS-2, ADI-R; SRS Repetitive Behavior; CCC-2; SCQ; RBQ; ICD-10; *IQ* (WASI, −2; Wechsler Word Reading and Operations; PPVT; DAS General Conceptual Ability; WISC-R, -III; Leiter NVIQ; *Theory of mind* (Strange stories; Frith-Happé Animations; ToM Task; Task of False Belief; Emotion Recognition Task; *Executive function* (Color-Word Interference; Letter and Number Sequential Memory; ADHD Rating Scale IV; Verbal Fluency; Working Memory; Planning; Flexibility; Amsterdam Neuro Task; London Tower; Luria Inhibition; Teddy Bear; Go/ Ngo; Digit Span; Affective Decision Delay Discounting; Wilding Attention Task; Vigilant Computerized Test; MAP; Forward Memory; Sustained Attention; Mazes Test; Set-shifting; Card Sort; Trail Making; A-not-B; Spatial Reversal Task; BRIEF; MPT *Central coherence* (CEFT; Hooper Visual Test; Pattern Construction; *Adaptive/ social behavior* (VABS; Facial Recognition; Identification Facial Emotion; Peer Victimization Self- and Parent-Report; SIS; *Co-occurring conditions* (SMFQ; CBCL; Aberrant Behavior Checklist; DISC, DISC-IV-P; Societal Functioning; SDQ; Beck Youth Inventory Self-Inform Anxiety, Depression; Spence Children Anxiety; ADHD Symptoms; CAPA; PONS; CBSQ; Mental Health and Medication *Academic functioning* (Reynell; PPVT; Grammar Test; Sentence; Word repetition; Letter Knowledge; Reading; DAS Basic Number Skills, Spelling, Word Reading; TAPS-AR; GORT-5; CTOPP RAN, NWR, Elision; TOWRE-2; CELF-RS; WIAT-RV; Early numerical competence: Subitizing, counting, magnitude, estimation; Mathematical domains: Number facts, problems, calculation, time; Letter Sound Knowledge; Phonological Awareness; Name Writing; NEPSY Phonological Memory; CELF-RS; YARK	Challenges in EF in autistic children without ID generally persist over time Regardless of measures, most studies indicated persistent and high rates of co-occurring mental health conditions/symptoms at follow up Parental affective disorder and family deprivation may be risk factors for co-occurring mental health conditions Performance on tasks for EE, ToM, and CC improve over time (inhibition and flexibility in particular) but impairments remain evident From childhood to adolescence, autistic children without ID seem to show reductions in anxiety, ADHD, ODD, and CD symptoms and slight increase in depression symptoms May be able to predict outcomes in middle childhood and adolescence through EF, autism features, and language skills
Pender (2020)^12^ Systematic review on autism features	No of studies 44 Sample size 20-9744 Age range 1-23 years Proportion of females reported in studies 0-51%	Follow-up period variable Design of included studies prospective	*Autism features – social communication and RRBs* (ADOS −1, −2, Toddler, -G, CSS, PL-ADOS; ADI, -R Social Communication; SRS Total, SCI, RRB; CARS; RBS-R; SCDC; SCQ; ABC)	Overall, some evidence for decreasing trend in social communication challenges but not in RRBs domain among individuals with autism diagnoses; decrease associated with higher V and NVIQ but not gender, adaptive functioning, language, or intervention ADOS CSS scores are stable over time due to being adjusted for development; a large number of studies using ADOS CSS found no significant changes and were less likely to find decreasing trend in outcomes Some evidence to suggest IQ predicts decrease in social communication challenges but not RRBs Some limited evidence that social challenges but not RRB traits reduce over time in autistic children When using LCGM, 4-class model (consistently high/low, increasing/ decreasing trajectories) was most reliable in characterizing data; supports inter-individual chronogeneity
Yeung (2024)^13^ Systematic review and meta-analyses on executive function	No of studies 14 Sample size of autistic participants 14-240 Age range 4.1-12.8 years (1 aging study, with mean age of autistic individuals 52.2 years) Mean baseline age of autistic individuals 5.0 to 14.2 years	Maximum follow-up period: <2 years (k=2), 2-3 years (k=7), ≥5 years (k=5) Sampling methods “often from hospitals and schools”	*Executive function constructs:* *- Planning* (London Tower; BRIEF parent report; BRIEF Norwegian parent report; *- Working memory* (LNST; BRIEF parent report; BRIEF Norwegian parent report; Digit span; Visuospatial n-back task; Wechsler based tests; Woodcock-Johnson III Test of Cognitive Abilities; Working memory test battery for children; N-back *- Inhibition* (CWIT Condition 3; BRIEF parent report; Go/Ngo; Mittenecker poing test; Stop-signal task; BRIEF Norwegian parent report; *- Shifting* (CWIT Condition 4; BRIEF parent report; TMT Condition 4; TMT; BRIEF Norwegian parent report; *- Initiation* (BRIEF parent report; BRIEF Norwegian parent report; *- Organization* (BRIEF parent report; BRIEF Norwegian parent report; *- Monitoring* (BRIEF parent report; BRIEF Norwegian parent report; *- Emotion control* (BRIEF parent report; BRIEF Norwegian parent report; *- Decision-making* (Iowa gambling task; *- Delay discounting* (Delay discounting test *- Letter fluency* (Divergent association task) *- Category fluency* (Groninger Intelligentie Test)	Autistic individuals have significantly poorer EF than TD individuals, but both groups demonstrate similar changes in EF over time Working memory most frequently studied, followed by inhibition; decision-making and delay discounting were the least frequently studied Most studies were focused on school-aged children

Abbreviations: ABAS-2: Adaptive Behavior Assessment System – Second Edition; ABC: Autism Behavior Checklist; ABCL: Adult Behavior Checklist; ADI-R: Autism Diagnostic Interview-Revised (IS: Insistence on Sameness factor (three items of ADI-R); ADOS: Autism Diagnostic Observation Schedule (CSS: Calibrated Severity Score; SA: Social Affect; RRB: Repetitive and Restricted Behavior); ASEBA: Achenbach System of Empirically Based Assessment; ASSQ: Autism Spectrum Screening Questionnaire; BDI-II: Beck Depression Inventory-II; BPS: Behavior Problem scale; BPVS-2: British Picture Vocabulary Scales, Second Edition; BSID: Bayley Scales of Infant Development; CAPA: Child and Adolescent Psychiatric Assessment; CASL: Comprehensive Assessment of Spoken Language core tests; CBCL: Child Behavior Checklist; CBSQ: Children’s Social Behavior Questionnaire; CDER: Client Development Evaluation Report, (Soc: rescaled sum of five social functioning items; Com: rescaled sum of three communication items); CDI: Children’s Depression Inventory; CEFT: Children Embedded Figures Test; CELF-RS: Clinical Evaluation of Language Fundamental-Recalling Sentences; CIS-R: Clinical Interview Schedule-Revised; CSI: Child Symptom Inventory; CTOPP: Comprehensive Test of Phonological Processing; CWIT: Color-Word Interference Test; DAS: Differential Abilities Scale; DBC: Developmental Behavior Checklist; DISC-IV-P: Diagnostic Interview Schedule for Children IV. Parent Version; DISC: Diagnostic Interview Schedule for Children; EAQ: Emotion Awareness Questionnaire; ECI-4: Early Childhood Inventory-4; EOWPVT: Expressive One-Word Picture Vocabulary Test; EVT-1: Expressive Vocabulary Test – First Edition; EXT: Externalising score; GORT: Gray Oral Reading Test; IS: Insistence on Sameness; LNST: Letter-Number Sequencing Test; MAP: Matching Associated Pairs; MBP: Maladaptive Behavior Problems score; MP: Merrill-Palmer; MSEL: Mullen Scales of Early Learning (DQ: Developmental Quotient; ELC: Early Learning Composite; NVIQ: Non-verbal IQ; VIQ: Verbal IQ; VR: Visual reception); MSEL: Mullen Scales of Early Learning, EL: Expressive language domain, RL: Receptive language domain; NCBRF: Nisonger Child Behavior Rating Form; NR: Not Reported; NWR: Nonword Repetition; PCQ: Parental Concerns Questionnaire; PDDBI: Pervasive Developmental Disorder Behavior Inventory; PLS-4: Preschool Language Scale-4 (AC: Auditory Comprehension; EC: Expressive Communication); PONS: Profile of Neuropsychiatric Symptoms; PPVT-3: Peabody Picture Vocabulary Test – Third Edition; RAN: Rapid Automatized Memory; RBQ: Repetitive Behaviors Questionnaire; RBS-R: Repetitive Behavior Scale-Revised; RRBs: Restricted and Repetitive Behaviors; RSM: Repetitive Sensory Motor factor (four items of ADI-R); SCARED: Screen for Child Anxiety Related Emotional Disorders; SCDC: Social and Communication Disorders Checklist; SCL: Somatic Complaints List; SCQ: Social Communication Questionnaire; SDQ: Strengths and Difficulties Questionnaire; SEFI-R: Social and Emotional Functioning Interview-Revised; SEQ: Social Emotional Questionnaire; SIS: Social Interaction Style Questionnaire; SMFQ: Short Mood and Feelings Questionnaire; SRS-1: Social Responsiveness Scale, First Edition; TAPS-AR: Test of Auditory Processing-Auditory Reasoning; TBP: Total Behavior Problems; TMT: Trail Making Test; TOWRE-2: Test of Word Reading Efficiency; VABS: Vineland Adaptive Behavior Scales (Soc: Socialization; Com: Communication; DLS: Daily Living Skills; EL: Expressive language; RL: Receptive language); VCI: Verbal Comprehension Index; VPT: Various psychometric tests; W/RQC: Worry/Rumination Questionnaire for Children; WASI-2: Wechsler Abbreviated Scale of Intelligence – Second Edition (FSIQ: Full-scale IQ; PRI: Perceptual Reasoning Index); WIAT-RV: Wechsler Individual Achievement Test-Receptive Vocabulary; WISC: Wechsler Intelligence Scale for Children; WJ-III-ACH-LWI: Woodcock Johnson – Third Edition Tests of Achievement: Letter Word Identification subtest; WPPSI: Wechsler Preschool and Primary Scale of Intelligence; YARK: York Assessment of Reading for Comprehension.

### Changes in diagnosis over time

Key longitudinal autism cohort studies (table 2) illustrate how the field’s understanding of autistic development has evolved. Early cohorts explored the prevalence and persistence of autism. For instance, the Special Needs and Autism Project (SNAP) in the UK used systematic screening and found that autism prevalence was higher than previously reported.^14^ The Early Diagnosis of ASD Study in the US examined shifts between autism diagnostic subtypes, which informed the adoption of a single diagnostic grouping, autism spectrum disorder, in the DSM-5.^15^ ^16^ The Longitudinal Study of Australian Children examined autism diagnostic stability over time and found that persisting diagnosis and late diagnosis were the most common categories upon repeated assessment, although some children’s parents reported they no longer had an autism diagnosis.^17^

**Table 2:** Key longitudinal cohort studies of autistic children and youth

Name	Follow-up	Sample n and characteristics	Measures	Key Findings
Gothenburg Cohort, Sweden Inception: 1980s	Followed for 13-22 years; follow-up occurred 1999-2002 (n=108)	Original cohort 120 participants Re-evaluated at age 17-40 (mean age 25.5)	DISCO IQ VABS Psychiatric-medical evaluation Global Assessment of Functioning Classified outcomes into good / fair / restricted but acceptable / poor / very poor (based on combinations of higher education/vocational training, living independently, friends/relationships)	85% continued to meet autism diagnostic criteria. Some shifts across autism subtypes 57% deemed to have a very poor outcome (21% poor, 13% restricted but acceptable; 8% fair; 0 good). Three lived independently but were socially isolated.^102^
Howlin et al, UK Inception: diagnostic assessment before age 16 years (1980s-90s)	Average age at follow-up 29.3 years (SD 8 years; range 21-48)	Initial sample of 79 Inclusion criteria: seen primarily for autism diagnostic assessment (mean age at diagnosis 7.2 years) Follow-up sample n=68; inclusion criteria: NVIQ ≥50; ≥21 years of age, 61 M and 7 F No race/ethnicity data provided	Adulthood: ADI to confirm diagnosis, measure social functioning, behaviors IQ British Picture Vocabulary Scale Neale Test of Reading Ability Schonell Spelling Test Parental questionnaire Reports from employers, other professionals, medical records Classified outcomes into Good / Fair / Restricted but acceptable / Poor / Very Poor (based largely on combinations of higher education/vocational training, living independently, friends/relationships)	12% of participants had a very good outcome; 10% good; 19% fair; 46% poor; 12% very poor. Having an IQ ≥70 predicted “better” outcome than <70. Within those with IQ ≥70, still heterogeneous outcomes.^21^
Early Diagnosis of ASD Study, US Inception: age 2 years	Age 3 *Age 5* (NC n=103; Chi n=11; DD n=22) *Age 9* (NC n 87; Chi n=68; DD n=17) *Age 19* n=120	NC: n=214 Chi: n=83 Non-spectrum comparison group, including language delay and GDD (DD; n=22) *Baseline characteristics:* ASD/PDD (%) *F*=14/11 White=68/72 Black=30/26 Limited speech=74/57 *Age 9:* ASD/PDD *F*=14/14 White=65/83 Black=31/17 Limited speech=28/0 Age 19: n=85 (92% M); higher loss to follow-up for Black families with lower education	Initial assessment: ADI-R, ADOS, clinical impression, VABS, MSEL (DQ) / IQ Age 9: ADI-R, ADOS, VABS, IQ (verbal and NV), clinical impression Age 19: ADI-R, IQ (verbal and NV), VABS	Greater diagnostic instability between PDD and ASD in ages 2-5 years *v* 5-9 years Verbal IQ (9 years): 4 trajectories – 2 with rapid increase and then slower improvement, 1 with little change, and 1 with consistent high needs NVIQ (9 years) more stable^16^ *Age 19*: divided group into ASD+VIQ <70 (n=53) ASD+VIQ ≥70 (n=24) “Very Positive Outcome” (no ASD+VIQ ≥70) (n=8) Group 1 – more likely to have taken meds and had early intervention; had differences present at age 2 consistent with other two groups. Groups 2 and 3 did not differ at age 2.^103^
Pathways in ASD, Canada Inception age: 2 years to 4 years 11 months Year: 2004	Baseline (T1; mean age 3.41 years) T2: 6 months (3.99 years) T3: 12 months (4.51 years) T4: Age 6 T5: Age 7 T6: Age 8 T7: Age 9 T8: Age 10 T9: Age 13 T10: Age 15	Inclusion criteria: aged 2 years to 4 years 11 months; recruited within 4 months of diagnosis Baseline n=421; 84.3% M Mean age at dx 38 months Mean age at enrollment ~40 months (No racial data; distributed across 5 Canadian sites) 6 year follow-up n=285 10 year follow-up n=187 children with complete data Family income: ≥$80 000 51.5%; $40-80k 26.1%; <$40k 17.2%. 70% White Born outside Canada: 27.1%	Baseline: VABS, ADOS, ADI-R, PLS-4, Merrill-Palmer (DQ/IQ), ABC T2: VABS, “autism symptom severity” (ADOS-CSS), ABC T3: months: VABS, ABC T4: VABS, ADOS-CSS, ADI-R, CBCL, PLS-4, ABC T8: VABS, ADOS, Family Background Information Questionnaire, CBCL, ABC T9-10: VABS, Participation and Environment Measure for Children and Youth (PEM-CY)	10 year trajectory results: 2 trajectories based on ADOS-CSS as outcome: continuously improving (27%) and improving then plateau (73%). Baseline “less symptomatic/higher functioning” measures accordingly predicted group membership; improving group had fewer participants from lower income homes.^104^ Later focus on timing of changes (chronogeneity) – turning points (school entry, age 9-10 years).^22^
Special Needs and Autism Project, UK Inception: 9-10 years old	T2: 14-16 years (criteria: autism + full-scale IQ ≥50) T3: Age 23	Started with total population cohort of ~57 000 and identified those with confirmed autism diagnosis (n=255) or likely (n=1515) T2: n=100 T3: n=121	Baseline: Stratified sample received comprehensive assessment with ADOS, ADI-R, SCQ, language, IQ CAPA Service use at follow up interview T2: IQ, ADOS, ADI-R, SRS, SDQ, CAPA, Profile of Neuropsychiatric Symptoms, suite of executive functioning measures, suite of Theory of Mind measures T3: employment outcomes, independence, anxiety and depression, QoL	70% had at least one mental health condition and 41% had ≥2.^18^ Adulthood: 36% in competitive employment, 54% had frequent contact with friends. 5% living independently, 37% require overnight care. Moderate/severe anxiety in 11%; moderate/severe depression in 12%. Subjective QoL similar to UK averages except social relationships. Living situation, employment, education, and physical health predicted by childhood IQ, autism traits, and adaptive scores. No predictors for mental health, friendships, other aspects of QoL.^19^
Avon Longitudinal Study of Parents and Children, UK Inception: 14 541 pregnant people in Bristol April 1991 to Dec 1992	6, 12, 15, 18, 24, 30 months Age 11 – autism diagnostic status Ages 12, 14, 16, 18, 21-25	13 971 children surviving to age 7	No single measure used to capture early development that was repeated at three time points, so not possible to measure trajectories. Abbreviated existing scales (Denver Developmental Screening Test, MacArthur CDI). Autism diagnoses made by MDT and clinical records reviewed by pediatrician. Adult measures of autistic traits, ADHD features, psychosis/schizophrenia, mental health	Autism signs in first 30 months – concerns about hearing and vision by 12 months; autism features + differences in feeding habits by 24 months.^105^ Autism and ADHD traits in adulthood (age 25 years) showed similar characteristics to childhood. Self-reported challenges in cognition, learning, and communication less pronounced in self-report than by parents.^106^
Longitudinal Study of Australian Children, Australia Inception: Birth cohort 2004 Kinder cohort (aged 4-5 years) born 2000	T1: 2004; assessed at two-yearly waves T7 (2016)	Two Australian representative cohorts of children T7 (2016) n=3381 (66%) respondents in Birth cohort (age 12–13 years) and n 3089 (62%) for Kinder cohort (age 16–17 years)	Parents asked about autism diagnostic status (among a list of conditions) every 2 years until age 12 (Birth) and 16 (Kinder) Socio-Economic Indexes for Areas Disadvantage Index and other demographics Parents asked about receipt of services PPVT IQ SDQ	Birth cohort (n=66) and Kinder cohort (n=73) diagnosed as autistic at one time point. 14% of Birth cohort and 26% of Kinder cohort no longer had reported autism diagnosis by T4. Persisting or late trajectories were most common.^17^ Frequent prescription of psychotropic medications to autistic children (>25% had polypharmacy).^20^
Yokohama Longitudinal ASD Birth Cohort, Japan	20 year follow-up	Born in northern Yokohama 1988-1996, diagnosed as autistic by age 7 years (n=278) 20 year follow-up cohort: mean age 24.6 (range 21-29) M:F ~3.5:1 Age 5 IQ 70.1 (25.4) 81% underwent early intervention	20 year follow-up data were extracted from medical records and interviews with psychologists. Applied Howlin’s composite outcome of overall social functioning based on work, independence, friendships. Also measured place of residence, work/education, time in activities per week, income, recreation/hobbies VABS, IQ, ABC	20 year follow-up: Psychosocial outcomes were very good (13.7%), good (25.0%), fair (31.0%), poor (25.6%), and very poor (4.8%) For those with IQ >50, outcomes not associated with IQ levels.^107^

ABC: Aberrant Behavior Checklist; ACSF:SC: Autism Classification System of Functioning: Social Communication; ADHD: attention-deficit/hyperactivity disorder; ADI/ADI-R: Autism Diagnostic Interview – Revised; ADOS: Autism Diagnostic Observation Schedule; ADOS-CSS: ADOS Calibrated Severity Score; AIM: Autism Impact Measure; ASD: autism spectrum disorder; CAPA: Child and Adolescent Psychiatric Assessment; CBCL: Child Behavior Checklist; Chi: Chicago sample; CP: cerebral palsy; DD: developmental delays group; DISCO: Diagnostic Interview for Social and Communication Disorders; DQ: developmental quotient; F: female; IQ: intelligence quotient; M: male; MDT: multi-disciplinary team; MSEL: Mullen Scales of Early Learning; NC: North Carolina sample; NDDs: neurodevelopmental diagnoses; NVIQ: non-verbal intelligence quotient; PDD: pervasive developmental disorder; PLS-4: Preschool Language Scales, 4th edition; PPVT: Peabody Picture Vocabulary Test; QoL: quality of life; RRBs: restricted/repetitive behaviors and interests; SCQ: Social Communication Questionnaire; SDQ: Strengths and Difficulties Questionnaire; SRS: Social Responsiveness Scale; T: time; UK: United Kingdom; VABS: Vineland Adaptive Behavior Scales; VIQ: verbal IQ

### Main outcomes

Overall, the outcomes across the reviews were highly variable and findings were mixed. Despite this heterogeneity, several consistent findings emerged. Among autistic individuals, higher levels of anxiety and depression in childhood significantly predict higher levels of these symptoms later in life.^10^ Autism features were generally stable over time among autistic children without intellectual disability,^11^ while “challenging” (externalizing) behaviors showed small but statistically significant declines in autistic children.^9^ Social communication tended to improve, while restricted and repetitive behaviors were more persistent.^12^ Although executive function challenges in autistic children and adolescents often persisted, there was evidence that they can improve with time and support.^13^

### Co-occurring conditions

As the cohorts age, the increasing relevance of co-occurring conditions has become apparent. Intelligence quotient (IQ), and relatedly, diagnoses of intellectual and developmental disability (IDD), were among the most common metrics in the cohorts, including at their inception. Because of this, IQ has been highly studied as a predictor of adult outcomes in autism. IQ scores in the IDD range in childhood were more consistently associated with “poorer” adult outcomes, but the findings for those with IQ ≥70 were more varied.

Mental health conditions become an increasing focus over time. The SNAP found high rates of co-occurring mental health conditions (70% had at least one mental health condition, and 41% had two or more) in their initial sample,^18^ and relatively high rates of moderate to severe anxiety/depression (11-12%) in their adult follow-up that were not predicted by childhood indices.^19^ Relatedly, the Longitudinal Study of Australian Children reported frequent use of psychotropic medication in their autistic sample, including polypharmacy in more than a quarter of participants.^20^

### Holistic outcome measurement

A common outcome classification was first used by a UK based group, combining ratings of the person’s occupation/education, friendships, and independent living into adult outcomes of “very good,” “good,” “restricted but acceptable,” “poor,” and “very poor.”^21^ While ratings in these categories have skewed toward the poorer outcomes, an interesting finding from the SNAP cohort was that subjective quality of life was similar to UK averages, with the exception of social relationships.^19^

The Pathways in ASD study in Canada employed assessments approximately every two years, starting at preschool diagnosis, to understand trajectories of autism features and adaptive functioning through to adolescence, identifying key inflection points, including school entry.^22^ The Pathways group adopted a strengths based measurement model through quantitative aspects (reframing of outcomes in relation to “doing well”, a person centered construct that incorporates both absolute and relative growth) and qualitative aspects (parent and teacher reported “best things” about the participants, including kindness, humor, and joyfulness).^23^ ^24^ ^25^ “Doing well” in subdomains of adaptive function, such as communication, social interactions, and activities of daily living, was associated with child level variables (such as cognitive and language level) as well as family level ones (such as household income, family functioning).^23^ This finding resonates with findings from other longitudinal cohorts, emphasizing that family and community level variables that comprise “social determinants of health” routinely considered in non-autistic populations are also fundamental to the early experiences and developmental trajectories of autistic children and youth.^26^

Overall, we found that longitudinal cohort studies in autism have developed alongside the autistic children and youth they have studied. These studies have been foundational to our understanding of autism. They are also inherently limited by the passage of time and create challenges when we attempt to apply our current conceptualization of autism to decisions made decades earlier at study inception. Notably, current research on outcomes in autism cannot support precise predictions about an individual child’s future. Outcomes are influenced by numerous non-autism factors: co-occurring developmental diagnoses and cognitive abilities, mental health conditions, family factors, and social determinants of health.

## Interventions for autistic children and youth

### Definition

The term “intervention” has become more contentious related to implications that it seeks to “cure” autism, as opposed to seeing autism as a neurotype within the neurodiversity paradigm.^27^ This tension has heightened with recent critical attention to the safety^28^ and ethics^29^ of some approaches. In this review, we consider meaningful interventions to be programs that directly and intentionally effect change for the purpose of enhancing skills and functioning, as opposed to seeking to make a person neurotypical. We also distinguish interventions from more general efforts to create a supportive environment for development, recognizing that such environmental modifications can also be included in interventions.^30^

### Types of interventions

A diverse range of intervention modalities have been applied to support skill acquisition and developmental trajectories in autistic children (table 3). Published research until the early 2000s predominantly focused on behavioral approaches with a therapist prompting and reinforcing target responses to specific structured inputs. Since then, approaches focus more on aligning goals and instructional methods to the developmental needs of the child, and to integrating learning opportunities into day-to-day experiences and involving key social partners, particularly parents (such as naturalistic developmental behavioral interventions).

**Table 3:** Intervention modalities for autistic children and youth

Intervention modality	Description
Behavioral intervention	Therapist-directed interventions based on operant learning theories, relying on behavior analytic techniques such as didactic instruction, prompting, shaping, and extrinsic reinforcement. Also referred to as applied behavioral analysis (ABA)
Developmental intervention	Interventions implemented according to a typical developmental sequence to facilitate foundational developmental milestones and skills and involve following the child’s lead
Natural developmental behavioral intervention (NDBI)	Interventions combining adult-led behavioral teaching methods with child-led routines, with a focus on teaching skills in a natural developmental progression using natural environments and rewards. Generally parent-led, with modeling and/or coaching by therapist
TEACCH Autism Program^106^	Specific intervention framework focusing on the learning strengths and preferences of the individual, utilizing a “structured teaching” approach to organize physical environments with activities, emphasizing visual supports and structured routines to promote independence and engagement
Sensory-based intervention	Interventions that involve exposure to sensory stimuli to address sensory processing differences in individuals. Examples include Ayres Sensory Integration,^109^ music therapy, and weighted blanket therapy
Animal-assisted intervention	Interventions mediated by the presence of an animal to improve social, emotional, and behavioral outcomes. Common examples include equine-assisted therapy or therapy dog interactions
Technology-based intervention	Interventions primarily delivered by electronic devices, computers, or other specialized technological hardware. Interventions in this category also include robot-mediated interventions for social skills, and virtual reality systems to simulate social environments
Physical activity based intervention	Interventions that utilize structured physical exertion, such as sports, mind-body exercises, or games to improve cognition, social skills, and motor function. The focus can range from individual exercising programs to games in group settings
Social skills	Interventions that use a structured curriculum to facilitate social interaction, emotional intelligence growth, and communication strategies, often in a group setting. Specific programs, such as the Program for Education and Enrichment of Relational Skills (PEERS),^110^ target making and keeping friends through lessons and role-play activities
Cognitive behavioral therapies	Psychological interventions to manage co-occurring mental health conditions, such as anxiety or depression. The focus is on identifying and working through negative thought patterns and behaviors, adapted for autistic youth (such as increased structure, visual aids)

Over the past 20 years, the number of clinical trials focused on older autistic children has increased exponentially, with a broader range of modalities evaluated, ranging from programs that focus specifically on social skills development, to technology based approaches such as using virtual reality systems to simulate social environments, and physical activity based programs that utilize structured activities such as sports and mind-body exercises.

We reviewed systematic reviews conducted in the past six years (2019-2025), identifying 46 that met our criteria, Although some reviews included a broader age range, most focused on either preschool (<6 years old, n=18) or older youth (n=28), and many of the newer intervention approaches were applied predominantly to the latter. Hence, we organize our summary by age group, highlighting key findings for each modality. Current understanding of how interventions impact the development of autistic children during early childhood has been greatly informed by meta-analytic work from Project AIM (Autism Intervention Meta-analysis; six of the preschool review articles), which is unique in its depth and scope, and will therefore be presented separately.

### Outcomes of early childhood interventions

#### Project AIM

This 2020 landmark systematic review and meta-analysis covered randomized controlled trials (RCTs) and quasi-experimental studies published to November 2017, with a focus on children younger than 8 years.^7^ It used a comprehensive quality assessment framework that stratified study findings based on random assignment, concealed assessment of outcomes and sources of bias, such as the caregiver mediating the intervention and reporting on outcomes. They reviewed seven intervention categories—behavioral, developmental, natural developmental behavioral intervention (NDBI), TEACCH Autism Program, sensory based, animal assisted, and technology based—although some interventions did not have enough studies with common outcomes to generate effect size estimates. When all study types were included, significant positive effects were found for behavioral, developmental, and NDBI interventions; however, when limited to RCTs, positive effects were reported only for developmental interventions and NDBIs. Further in depth analyses of expressive and receptive language outcomes^31^ and NDBI outcomes^32^ were also reported for the original Project AIM dataset.

In 2023, the Project AIM group updated the systematic review and meta-analysis, adding studies published to November 2021.^33^ As in the prior review, there were not enough studies of animal assisted interventions, cognitive behavioral therapy, TEACCH, music therapy, and sensory integration/sensory based interventions to generate summary effect size estimates on any outcome. The differences in findings between the two reviews (restricted to RCTs) are summarized in table 4.^7^ ^33^

**Table 4:** Comparison of intervention study findings from two meta-analyses by Sandbank and colleagues (2020, 2023)

Intervention type	Sandbank et al 2020^7^				Sandbank et al 2023^33^			
	All RCTs		RCTs without caregiver report		All RCTs		RCTs without caregiver report	
	Outcome (No of studies)	Effect size (95% CI)	Outcome (No of studies)	Effect size (95% CI)	Outcome (No of studies)	Effect size (95% CI)	Outcome (No of studies)	Effect size (95% CI)
Behavioral	—	—	—	—	Social communication (n=9)	0.54 (−0.24 to 1.32)	—	—
					Social emotion or challenging behavior (n=10)	0.58 (0.11 to 1.06)*	—	—
Developmental	Social communication (n=11)	0.27 (0.05 to 0.48)*	Social communication (n=11)	0.31 (0.09 to 0.54)*	Social communication (n=14)	0.28 (0.12 to 0.44)*	Social communication (n=14)	0.31 (0.14 to 0.39)*
Natural developmental behavioral intervention (NDBI)	Cognitive (n=7)	0.18 (−0.18 to 0.46)	Cognitive (n=7)	0.18 (−0.10 to 0.46)	Adaptive (n=11)	0.23 (0.02 to 0.43)*	—	—
	Language (n=16)	0.21 (0.01 to 0.41)*	Language (n=14)	0.17 (−0.05 to 0.38)	Cognitive (n=13)	0.18 (−0.02 to 0.38)	Cognitive	0.19 (−0.02 to 0.39)
	Play (n=6)	0.33 (0.15 to 0.54)*	Play (n=6)	0.33 (0.15 to 0.54)*	Autistic features (n=17)	0.38 (0.17 to 0.59)*	Autistic features	0.44 (0.20 to 0.68)*
	Social communication (n=17)	0.42 (0.23 to 0.62)*	Social communication (n=13)	0.47 (0.26 to 0.67)*	Language (n=26)	0.16 (0.01 to 0.31)*	Language	0.13 (−0.04 to 0.30)*
					Play (n=8)	0.19 (0.02 to 0.36)*	—	—
					RRB (n=7)	−0.01 (−0.32 to 0.31)	—	—
					Social communication (n=32)	0.35 (0.23 to 0.47)*	Social communication	0.36 (0.23 to 0.49)
Sensory†	Language (n=17)	0.28 (−0.19 to 0.76)	Language (n=6)	0.28 (−0.31 to 0.86)	—	—	—	—
TEACCH Autism Program	—	—	—	—	—	—	—	—
Technology-based	Social communication (n=8)	0.06 (−0.19 to 0.30)	—	—	Language (n=9)	0.21 (−0.13 to 0.55)	Language (n=8)	0.26 (−0.14 to 0.66)
					Social communication (n=17)	0.33 (0.02 to 0.64)*	Social communication (n=13)	0.20 (−0.01 to 0.41)*
					Social emotion or challenging behavior (n=8)	0.57 (0.04 to 1.09)*	Social emotion or challenging behavior (n=7)	0.64 (−0.07 to 1.36)
Other	—	—	—	—	—	—	—	—

RRB: Repetitive and Restricted Behavior.

* P<0.05

† There was a single category of sensory based interventions in Sandbank et al 2020, whereas in Sandbank et al 2023, these were separated into multiple categories (such as sensory-integration therapies analyzed separately) reducing the total numbers; none of the categories of sensory-related interventions met the threshold of 5 clinical trials reporting an outcome domain to be included in the summary tables.

New findings from the 2023 review included improvement in social-emotional/dysregulated behavior with behavioral interventions, and for NDBIs, improvements in adaptive behavior, reduced levels of autistic features, and improvements in language. For technology based interventions delivered by computer or tablet or by robots, there were improvements in social communication as well as social-emotional/challenging behavior. When effects were further restricted to exclude caregiver or teacher reported outcomes, significant effects were detected only for developmental interventions and for NDBIs on social communication, and for NDBIs on autistic features. When effects were then restricted to exclude those at high risk of detection bias, only one significant summary effect was estimated—NDBIs on measures of autistic features.

Project AIM also assessed the relationship between intervention effect size and intensity, assessed three ways: daily intensity, overall duration, and cumulative intensity.^34^ This secondary meta-analysis of 144 studies that included 9038 children (mean age 49.3 months) did not reveal any association between intensity and effect size within intervention type. Results did not support a minimum threshold of intervention intensity needed for effect. The authors recommended that clinicians should work with families to calibrate individualized support for autistic children at an intensity that leads to individual benefit without compromising on activities and routines in home, educational, and community settings.

#### Additional systematic reviews

An additional 12 systematic reviews focused on preschool children were identified, eight of which included meta-analyses (Appendix 2). When compared with Project AIM, study inclusion criteria for most other reviews were broader, and meta-analyses included less stringent inclusion criteria and a more diverse set of interventions, although most reviewed fell into the broad categories of behavioral, developmental, and NDBI. The NDBI interventions (either considered alone^35^ or with a broader range of “play based” approaches^36^) were associated with significant improvements in language and social communication skills, consistent with Project AIM findings.^7^ ^33^

A meta-analysis of interventions for children younger than 24 months with early features or confirmed autism diagnoses (these were mostly NDBI models) indicated that gains were limited to parent reported adaptive skills.^37^ A second review on this age group reported that such gains vary across studies, but improvements in parents’ interaction strategies were more consistent.^38^ Reviews did not consistently stratify findings by child or intervention model (beyond broad modality) to inform individual-level planning. An exception is a recent meta-analysis, which indicated that children with more advanced baseline cognitive, language, and adaptive function demonstrated greater effects across approaches, and that intensity and duration of intervention, rather than intervention modality, influenced outcomes.^39^

### Outcomes of interventions for school-aged autistic children

Interventions were broader than for preschool children (Appendix 3) and included behavioral (inclusive of NDBI),^40^ animal assisted,^41^ ^42^ exercise,^43^ ^44^ ^45^ cognitive behavioral therapies for co-occurring mental health conditions,^46^ ^47^ ^48^ expanded application of technology based approaches (including virtual reality^49^ and robotics^50^), as well as specific social skills interventions including Program for the Education and Enrichment of Relational Skills (PEERS)^51^ (table 3).

While small to moderate summary effects were reported for many of these interventions, most included studies were limited by design biases, including confounding between intervention delivery and assessment, lack of blinding, and outcome measurement only in the context of the intervention. Some reviews spanned a broad age range (some from infancy to adulthood) or attempted to synthesize findings across highly disparate intervention approaches, making them difficult to interpret. NDBIs and other behavioral interventions were associated with similar outcomes as reported in younger children (such as improved language and adaptive function).^40^ There were promising findings from a heterogenous group of interventions targeting friendship development that improved social functioning and cultivated meaningful relationships.^52^ A meta-analysis of the PEERS intervention in autistic adolescents found large increases in social knowledge as well as parent rated social skills,^53^ findings that an additional meta-analysis reported applied to diverse international settings.^51^ There was some evidence for small to moderate effects of exercise on communication skills as well as executive function, especially with physical activities that included a social and/or mindfulness component.^45^ Some evidence suggested that executive function and social skills could be supported by immersive virtual reality experiences.^49^

Overall, there is an increasingly robust body of evidence guiding interventions from early in development to the adolescent years. However, despite the rapid growth of the number of RCTs, intervention effects based on meta-analyses are generally small to medium in size, may be inflated by study biases, and are generally not related to intensity of intervention. There is evidence that the development of language, social communication, and other adaptive skills can be enhanced through interventions early in childhood, particularly using NDBI models, although estimates of effects are attenuated when the most stringent criteria for study design are applied. Outcomes for interventions designed for older youth have included a greater emphasis on mental health and social relationships and physical activity. Interpretation of findings from these studies is limited by similar design limitations as those of younger children, including outcome assessment only occurring in the context of the intervention and/or evaluation that focuses on respondents that are directly engaged in the programs. 

## Insights from autistic people

### Reviews of community based participatory research methods in autism

Reviews of community based participatory methods in autism research reveal the growing importance of inclusion of autistic voices. Indeed, one of the earlier reviews, analyzing engagement of various interest holders in biomarker research, found that studies had only included parents, not autistic people themselves.^62^ An additional early review noted limitations in the depth of reporting of autistic partner involvement.^63^ A key development for the inclusion of autistic people in research was the publication of the Academic Autism Spectrum Partnership in Research and Education (AASPIRE) Practice Based Guidelines for Inclusion of Autistic Adults as Co-Researchers and Study Participants.^64^ These guidelines set out best practices for engagement with autistic people, with the aim of improving the validity and applicability of the resulting research.

### Acceptance and inclusion of autistic people

Studies on the lived experience of autistic individuals reveal insights into how community, contextual, and systems level inputs affect their function, wellbeing, and participation. In one survey (n=144), supportive communities were consistently associated with improved mental health and social outcomes in autistic individuals, as they facilitated acceptance, belonging, and self efficacy.^65^ One qualitative study described the relationship between family autism acceptance and increased resilience and decreased stress among autistic people.^66^

Schools can be a pivotal environment for autistic youth, with a systematic review (256 parents/carers and 101 educational staff) linking inclusive education settings with improved academic and social outcomes.^67^ One qualitative study interviewed 10 autistic adults who described high school as a “turning point” associated with increased confidence and accepting their autistic identity.^68^ Qualitative studies interviewing autistic adolescents have also highlighted the importance of teachers leveraging autistic students’ strengths and interests,^69^ as well as access to safe spaces and autonomy for decisions in the school day.^70^ Opportunities for friendship are also important: an observational study of over 300 autistic and non-autistic youth showed an association between positive friendship quality and lower depressive symptoms.^71^

## Influence of contextual factors

### Sex/gender and autism

#### Autistic individuals assigned female at birth (AFAB)

Recent reviews focused on autistic individuals AFAB have shed light on the frequent health inequities faced by this population. Much of the research for this group has come from qualitative work and administrative data, with comparatively fewer prospective cohort studies.^72^ A 2023 review exploring the challenges with autism identification and support for autistic people AFAB attributed their delayed, mistaken, and missed diagnoses to a complex interplay between social (such as clinician biases, masking), structural (such as insufficient workforce capacity for care and diagnosis), and research and policy level barriers (such as poor representation of autistic AFAB groups in research).^73^ These diagnostic gaps delay access to support and were linked to poorer outcomes such as low self esteem, traumatic experiences (such as social exclusion and alienation, discrimination, sexual harm), and persistent mental health challenges.^73^

A 2022 review of qualitative studies involving autistic people AFAB diagnosed in adulthood further corroborated the multi-level barriers they face in receiving autism diagnosis and care.^74^ It also highlighted the critical role of timely autism diagnosis in identity building, accessing supports, and fostering better life experiences overall. Furthermore, a 2020 scoping review on the physical health of autistic individuals AFAB described the significant co-occurring physical health challenges they face, such as epilepsy, in addition to reproductive (such as menstrual challenges, endocrine related issues) and metabolic conditions including obesity and diabetes.^72^ Autistic individuals AFAB also have a greater risk of premature mortality due to suicide than autistic people assigned male at birth (AMAB), as reported in a population based study.^75^ The 2023 review also revealed that autistic children and youth AFAB face greater social exclusion among their peers, and in adulthood, are less likely to have consistent involvement in postsecondary education and employment compared with autistic individuals AMAB. Existing systemic barriers (such as racism, poverty, stigma) further compound these inequities and disparities in care and outcomes for autistic individuals AFAB with intersectional identities.^73^

#### Autism and gender diversity

This is a newer relatively understudied area in autism outcomes research. Two recent meta-analyses of observational studies (including 3894^76^ and 8662^77^ participants) found that 7-11% of autistic individuals identify as gender diverse,^76^ significantly higher than in the general population.^77^ A qualitative study of autistic youth who had experienced gender dysphoria and their parents identified distress as a prominent theme, as well as the conflicting identities and needs between the two groups, with youth focused on gender needs and parents focused on autism needs.^78^ Consensus based guidelines recommend that treatments must address both gender diversity and autism, and suggest that assessment and treatment phases may overlap as the youth gains more insight.^79^ These guidelines also highlight the heightened risk for bullying, exploitation, and violence for this group, as well as risks of stigma and discrimination in education and employment. A panel of experts on autism and gender diversity, including people with lived experience, identified the need to acknowledge and understand the barriers faced by gender diverse autistic people, promote access to gender affirming care, and embed neuro-affirming principles within gender care.^80^

### Culture, race/ethnicity, language, and migration status

A growing number of studies have explored the complex interplay between autism and factors such as culture, race/ethnicity, language, and migration status/history, often employing qualitative methods to capture perspectives and experiences. A 2021 systematic review, including both quantitative, qualitative, and mixed methods studies, examined parental perceptions of autism within American Latinx and Black communities, revealing that cultural beliefs significantly influence the understanding and approach to autism, and emphasizing the importance of culturally tailored interventions to improve engagement and outcomes.^81^

A 2024 systematic review of various study types (quantitative, qualitative, and mixed methods) focused on factors affecting access to autism services among culturally and linguistically diverse families.^82^ Their results identified that language barriers, cultural stigma, and lack of culturally competent providers hinder service utilization, highlighting the need for systemic changes to enhance accessibility. A 2022 scoping review examined barriers to healthcare access for minority language speakers with neurodevelopmental conditions, including autism, and found that language discordance between patients and providers, along with a lack of interpreter services, significantly impedes effective care delivery.^83^

Experiences of immigrant and refugee care givers of autistic children in Canada were explored in a 2023 review (including quantitative, qualitative, and mixed methods studies).^84^ Their study identified unique challenges, including navigating unfamiliar healthcare systems and cultural differences in perceptions of disability, which impact service access and utilization. While this is a burgeoning area of study, these studies collectively highlight the critical need for culturally and linguistically responsive practices in autism services.

## Research from low and middle income countries (LMICs)

In recent years, autism intervention research in LMICs has increasingly focused on scalable, community delivered models that address both access and equity gaps in early detection and treatment. A pivotal contribution to this field is a 2021 annual research review that synthesized over 30 reviews to present a “Theory of Change” framework for achieving universal health coverage for young autistic children in LMICs.^85^ The review emphasizes task-shifting strategies—where non-specialists such as community health workers or early childhood educators deliver interventions—as critical to overcoming severe shortages in specialist providers. Authors highlight the success of parent mediated interventions such as the Parent-mediated Autism Social communication Intervention for non-Specialists (PASS) and its enhanced version, PASS Plus, which have demonstrated acceptability, feasibility, and promising outcomes in India and Pakistan when delivered by lay health workers using structured video feedback and caregiver coaching techniques.

Other reviews have identified key factors for autism interventions in LMICs. One 2022 meta review of systematic reviews found that, although evidence based programs are well established in high income countries, most interventions evaluated in LMICs were small scale, pilot level studies, often requiring more rigorous study designs.^86^ Nonetheless, interventions with active parent involvement and behavioral components showed consistent promise. However, implementation barriers—such as financial constraints, lack of policy support, and cultural mismatches between intervention models and local norms—were common and typically underreported.

A 2017 scoping review similarly underscored the limited research base and underdeveloped infrastructure for intervention in sub-Saharan Africa.^87^ Small scale trials in Kenya, South Africa, and Uganda piloted the use of caregiver coaching, adapted screening tools, and community health worker training with early indications of feasibility. Five RCTs in India and Pakistan were included in a review on parent mediated interventions, albeit with small samples and limited outcome reporting.^88^ In many LMIC contexts, stigma, spiritual beliefs, and systemic exclusion can reduce families’ engagement with services, highlighting the importance of culturally grounded approaches that actively include underrepresented groups and center family experiences.^87^

Together, these studies point to a converging consensus: autism interventions in LMICs must prioritize equity, community ownership, and adaptability. The strongest evidence to date supports parent mediated interventions delivered through task sharing models, supported by simple, open access screening tools, and embedded within existing health and early education systems.

## Clinical implications

Research on interventions in autism has failed to identify one-size-fits-all therapeutic modalities or intensity levels. Binary descriptions of whether an autism intervention is “evidence based” are outdated and reductive, failing to consider the many facets of study rigor, bias, and developmental targets. Clinicians may feel helpless as to how to advise families in this situation.

Authors of a 2020 editorial propose a framework to help families select interventions: informing families about a range of developmental supports; tailoring intensity recommendations to the needs of the child and family; and encouraging integrated multidisciplinary supports.^95^ An additional framework adds that clinicians should consider whether the intervention dose is “scientifically plausible, practical to deliver, desired by the child and family, and defensible when considered in relation to all available options and the individual preferences and characteristics of the child and family.”^96^ Our results affirm these approaches and further emphasize that shared decision making for therapeutic approaches should incorporate other aspects of the child’s identity (such as gender), as well as the family’s identity and background (such as race/ethnicity, culture, and language).

Clinicians should also be mindful of the many contributors to quality of life for autistic people outside of autism-focused interventions. In the traditional medical context, this includes timely identification and management of co-occurring conditions. Autistic children and youth may present with a wide range of strengths and support needs across developmental domains. As such, effective care often involves coordinated medical, behavioral, educational, and psychological interventions tailored to the individual. Family level understanding and acceptance of autism creates a safe “home base” for the autistic person. Applying an intersectional lens, autism can interact with other marginalized identities and social determinants of health, creating cascading threats to wellbeing. Clinicians need to be attuned to these risks and their impacts, as well as being willing to advocate for their individual patients within too often oppressive systems of care.

## System and policy implications

This review reinforces the need for policies that recognize the heterogeneity of autism and support flexible, family centered, and inclusive service systems. Current funding and service models often favor prescriptive approaches to intervention delivery, prioritising quantity (such as hours of therapy) over quality, fit, or family priorities.^59^ Policies should support a range of developmental and support options, ensuring families can access services that are responsive to their child’s strengths, needs, and lived context.^56^ While these principles are currently embedded in some guidelines, policy changes and implementation science are necessary to ensure they reach children and families.

There is also a need to address systemic inequities in access to autism services. Families who are racialized, low income, live in rural areas, and are linguistically diverse often face structural barriers that lead to delayed diagnosis, reduced access to services, and poorer outcomes.^97^ Policy frameworks should center equity by promoting service models that are culturally safe, trauma-informed, and community based.^59^ ^98^ Investment in navigational supports and public education can help reduce disparities and empower families.

Developmental trajectories are influenced not only by clinical interventions but also by access to inclusive education, support for caregivers’ wellbeing, and comprehensive mental health and social services. Therefore, autism policy must go beyond the healthcare sector and intentionally foster coordination across education, early childhood, and social care systems.^59^ ^99^ Finally, ongoing engagement of autistic people and their families in shaping and assessing policy is vital to ensure that decisions remain meaningful, transparent, and grounded in lived experience.

## Emerging interventions

Accumulating evidence continues to refine established intervention models through the identification of moderators and mediators of intervention response. For example, caregiver strategy use has been shown to correlate with children’s functional gains across NDBIs,^54^ while characteristics such as baseline language ability, cognitive level, and family engagement increasing inform individual treatment planning.^39^ Although biomarker based stratification remains rare in non-pharmacological trials, emerging studies are beginning to link behavioral intervention outcomes with neurobiological or genetic markers.^55^ This convergence between developmental science and translational research offers a way of identifying subgroups who may benefit from specific types or intensities of behavioral intervention. While pharmacological and biomedical treatments remain beyond the scope of this review, these same precision principles are increasingly being applied to non-pharmacological domains to guide individualized, holistic care across development.

## Guidelines

We reviewed several published guidelines for interventions for autistic children and youth. Recent guidelines from the UK and Australia included exemplary reviews of the published literature as well as co-design with people with lived experience. The Australian guidelines included principles for selecting and delivering supports, collaborative goal setting, and monitoring safety and wellbeing during intervention.^56^ Authors recommended an individualized, child and family centered approach that is strength based and neurodiversity affirming, and that addresses the continuum of functional needs and areas of skill development. Similarly, the UK guidelines emphasize local access to coordinated services, involvement of multiple disciplines, promoting functional adaptive skills, supporting access to leisure, and supporting families.^57^ Neither of these guidelines endorse specific intervention models; rather, the application of principles to support evidence based practice and informed decision making. National guidelines from New Zealand,^58^ Canada,^59^ Spain,^60^ and the US^61^ also articulate principles of intervention without recommending specific treatment models.

## A holistic model of a “good life” for autistic children and youth 

**Fig 1:**
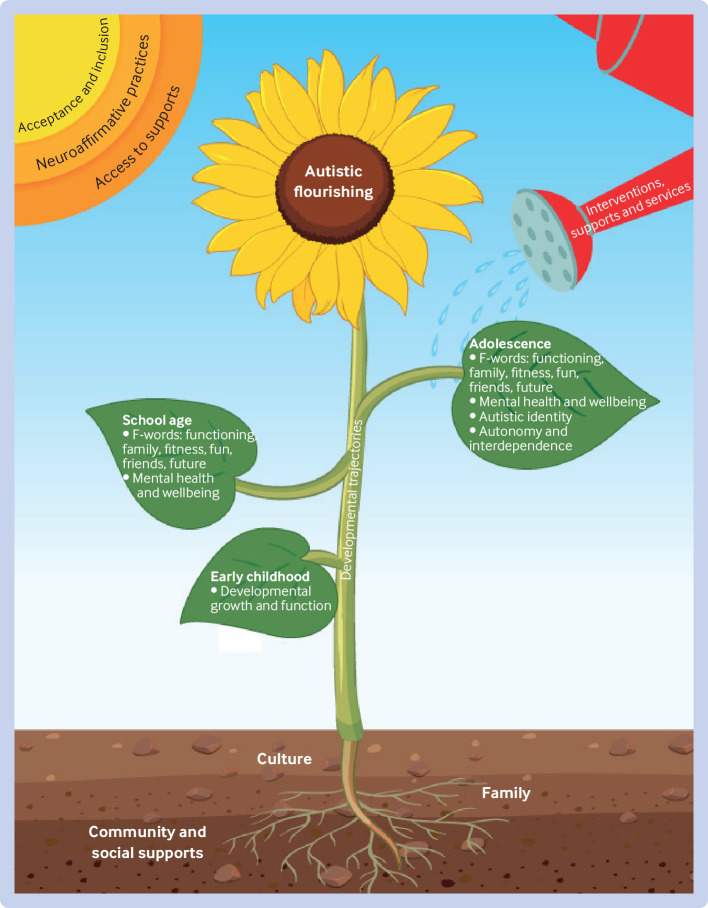
A representation of autistic “flourishing” We have represented a child’s growth using a flower, also representing the concept of autistic “flourishing.”^91^ The soil represents where the child is situated, including their family, culture, and community/social supports. The central stem indicates the child’s developmental growth. Leaves for each of Early Childhood, School Age, and Adolescence contain key features of these life stages, including the F-words of childhood disability.^90^ Interventions, supports, and services are represented by the watering can, indicative of the fact that the “dosing” is based on the child’s needs and environment. Finally, the sunshine represents an ongoing environment for growth and flourishing, including acceptance and inclusion, neuro-affirmative practices, and access to supports. Original illustration by Nick Abecassis.

This review aimed to summarize the state of the science with respect to outcomes for autistic children and youth, integrating concepts of intersectionality and affirmation of neurodivergence. Contributors to a “good” life in autism far exceed the core features of the condition. We have represented these varied contributors and indicators of autistic “flourishing” in figure 1. We have drawn from three important frameworks to inform this conceptualization. First, the “F-words in childhood disability” encompass a holistic view of domains that contribute to a good life: function, family, fitness, fun, friends, and future.^90^ Second, Pellicano and Heyworth described the “Foundations of Autistic Flourishing” in autism research.^91^ Translated to clinical practice, relevant aspects of this framework include promoting autistic wellbeing, amplifying autistic autonomy, paying attention to everyday experiences, acknowledging autistic people’s contexts, and working in partnership with autistic people and their families. Third, the sunshine in our model references neuro-affirming practices. Many of the initial descriptions of neuro-affirming care have identified language and practices that should be avoided, seeking to recognize past harms and avert these going forward.^92^ It is also important to frame neuro-affirming care in a positive sense. A framework from Jane offers several key concepts, which are embedded throughout our model: intersectionality, respecting autonomy, presuming competence, validating differences, rejecting neuronormativity, reframing expectations, promoting self advocacy, prioritizing lived experience, nurturing positive self identity, adapting systems and environments, and honoring all forms of communication.^93^ The figure depicts that trajectories are shaped by individual differences, contextual factors (including social determinants of health not specific to autism) as well as interventions.^26^

## Gaps in research

### Methodology considerations

Existing evidence from longitudinal cohorts and intervention studies has provided valuable insights into the lifespan needs of some autistic people and has left other areas unexplored. Autistic people AFAB and those with diverse gender identities are not well represented in existing cohorts, which is likely due to delayed clinical and societal recognition of how autism presents in these groups.^73^ Although many groups made efforts to recruit samples reflective of their populations, more work is needed to understand the features and experiences of autism in racially and socioeconomically diverse groups.

In the intervention literature, the Project AIM authors (2024) have raised concerns that selective exclusion of null and negative results from the literature, and thus from meta-analyses, can lead to positive bias in estimated summary effects and erroneous conclusions about what practices are evidence based.^94^ Other work from the Project AIM group reported that only 11 of the identified 150 studies included safety monitoring, which should be customary in measuring autism interventions.^28^ Notably, few of the included trials we reviewed focused specifically on children with more severe or profound developmental delays or disabilities, highlighting the limited evidence guiding support to this subgroup of autistic children.

More work is needed to understand the contributions of interventions and other services across the lifespan, with increasing focus on outcomes that are aligned to individual goal setting, occupational roles, and quality of life for both the child/youth and their family. The composite outcome measures employed in many studies include factors that have traditionally been conceptualized as part of a “good” life; however, the values espoused therein reflect highly Westernized and colonialist values in line with the traditional medical model of disability. Adding necessary texture and granularity to our understanding of the lives of autistic children, youth, and young adults will require studies that are informed by first person perspectives including diverse racial/ethnic and gender identities and representing broader global settings. Individuals with higher support needs must be considered within the group of diverse interest holders. Representation of this group requires reconsideration of study inclusion/exclusion criteria, thoughtful approaches to accommodation,^64^ and inclusion of trusted care partners to represent those for whom participation remains inaccessible.

### Future research

This review highlights several important directions for future research to improve understanding and support for autistic children and adolescents. First, there is a need for more longitudinal studies that follow autistic individuals into adulthood, particularly those from underrepresented groups, including racialized communities, gender diverse populations, and individuals with higher support needs. Expanding the inclusivity of study populations is essential to producing more generalizable and equitable findings. Larger datasets are needed to include and analyze all potential predictors of wellbeing, allowing for precision outputs that can support individualized anticipatory guidance.

Second, future research must move beyond binary or deficit based outcome measures to better reflect holistic understandings of autistic development that include strengths. This includes assessing wellbeing, identity formation, relationships and social connection, and participation in meaningful life activities. Studies that consider environmental influences, such as inclusive education, family acceptance, and access to affirming services, are needed to provide a more complete picture of the factors that support positive developmental outcomes.

A key priority of advancing this field requires embedding autistic co-design as a standard research practice. This approach involves autistic people as active partners in shaping research questions, methods, and outputs and is essential to producing meaningful, applicable knowledge. Studies show that autistic co-design enriches research outcomes by incorporating the lived experience and unique insights of autistic individuals, which may reveal challenges that non-autistic researchers alone may not recognize.^100^ Importantly, co-design processes need to account for additional time investment and the need for clear communication, as well as autistic individuals’ sensory and communication preferences.^64^ ^101^ While more resource-intensive, these efforts result in richer, more impactful research.

## Conclusions

This review underscores the complexity and heterogeneity of developmental trajectories and outcomes among autistic children and adolescents. A central finding is that there is no single pathway nor a one-sized-fits all therapeutic approach. Instead, effective clinical management requires personalized, flexible strategies that are responsive to the unique strengths, needs, and contexts of each child and their family. What is increasingly evident is that a “good life” for an autistic person is an individualized construct, rooted in a person’s family context, community, and culture. Moving forward, research and practice must center autistic voices in defining meaningful outcomes and continue to advance approaches that support holistic wellbeing across development. Clinicians play a vital role in this shift by applying a strength based, individualized lens to care, working in partnership with families to define and support outcomes that are not only clinically informed but also personally and contextually meaningful. This includes recognizing goals that reflect the broader dimensions of quality of life, identity, and participation, as defined by the individual and their family.

**Glossary of terms**

*Adaptive skills:* A collection of developmental skills contributing to participation in daily life.
*Autism features:* Individual characteristics, which may or may not be apparent to others, associated with autism diagnostic criteria.
*Executive function:* A collection of mental processes (inhibition, working memory, and cognitive flexibility) supporting planning, problem-solving, and goal-directed behavior.^111^
*Externalizing behavior:* Outward-directed actions that can interfere with an individual’s participation and may pose safety concerns.
*Intervention:* In this paper, we define meaningful interventions as those that directly and intentionally effect change to enhance skills and functioning.
*Late diagnosis:* Autism diagnosis occurring outside of the early childhood years, such as in adolescence or adulthood.
*Neurodiversity:* Referring to the neurological diversity present across human population.^112^
*Persisting diagnosis/autism diagnostic stability:* Continuing to meet criteria for an autism spectrum disorder diagnosis over time.
*Restricted and repetitive behaviors:* A domain of autism spectrum disorder diagnostic criteria^113^ containing stereotyped/repetitive motor movements, use of objects, or speech; insistence on sameness; interests that are unusual in their topic or intensity; and sensory differences (hypo/hyper-reactivity, aversions to sensory input, sensory-seeking behaviors).
*Social communication:* A domain of autism spectrum disorder diagnostic criteria3 containing challenges in social-emotional reciprocity (back-and-forth interactions); differences in non-verbal communication; and challenges in developing and maintaining relationships.

**How patients were involved in the creation of this article**

Our team of authors included an autistic researcher and two researchers who are parents of autistic children. All were involved throughout the generation of the article; specifically, the autistic co-author contributed the section on autistic perspectives.

**Research questions**

- What factors influence developmental trajectories and outcomes among autistic children and adolescents?
- What interventions positively influence developmental outcomes among autistic children and adolescents?
- How do contextual factors, such as sex, gender, culture, and race/ethnicity influence autistic development and outcomes?

## Web Extra

Extra material supplied by the author

Appendix 1: Search terms used in literature search

Appendix 2: Systematic reviews of intervention studies for preschool children

Appendix 3: Systematic reviews of intervention studies for school-aged children and adolescents
