# Supplementary material for: Advances in supporting development in autistic children and youth
Source: BMJ. 2026 Jun 10;393:e086562. doi: 10.1136/bmj-2025-086562 (PMC13250720; doi:10.1136/bmj-2025-086562)
Supplement: Supplementary file 1 — Appendix 1: Search terms used in literature search [file penm086562.w1.pdf]

Appendix 1: Search Terms

Longitudinal Cohort Studies:We searched for recent systematic and scoping reviews on observational longitudinal studies involving autistic children and youth using the following search terms: (autis* OR asperger* OR asd OR pdd OR pervasive developmental disorder) AND (longitudinal* OR trajector* OR follow up OR cohort OR prospective) AND ((systematic OR scoping) AND review). The search was restricted to studies published between Jan 1 2014 and May 31 2025.

Intervention studies: We searched for systematic reviews on intervention studies involving autistic children and youth using the following search terms: autis* OR asperger* OR asd OR pdd OR pervasive developmental disorder) AND ((intervention OR therapy OR teach* OR treat* OR program*OR package) AND (assign* OR control group OR BAU OR “wait list” OR RCT OR random* OR quasi OR “treatment group” OR “intervention group” OR “group design” OR trial)) AND ((systematic OR scoping) AND review). The search was restricted to publications from Jan 1 2019 to May 31 2025.

Autistic perspectives: We searched for systematic or scoping reviews on autistic perspectives in autism research using the following terms: ((autis OR asperger OR asd OR "pervasive developmental disorder" OR pdd))** AND (community-based participatory research)** AND ((systematic OR scoping) AND review). The search was restricted to studies published between Jan 1 2014 and May 31 2025.

Sex/gender: We searched for systematic or scoping reviews on (autis* OR asperger* OR asd OR pdd OR pervasive developmental disorder) AND (gender OR gender diverse OR sex OR afab OR assigned female at birth OR amab OR assigned male at birth OR transgend* OR non-binary OR gender identity) AND ((systematic OR scoping) AND review). The search was restricted to studies published between Jan 1 2014 and May 31 2025.

Marginalized communities: We searched for systematic or scoping reviews on intervention studies involving marginalized communities using the following search terms: ((autis OR asperger OR asd OR "pervasive developmental disorder" OR pdd))** AND ((intervention OR therapy OR teach OR treat OR program* OR package) AND (assign* OR “control group” OR BAU OR “wait list” OR RCT OR random* OR quasi OR “treatment group” OR “intervention group” OR “group design” OR trial))** AND((BIPOC OR “Black child” OR “Indigenous child” OR “racialized child*” OR “ethnic minorit*” OR “culturally diverse” OR “minoritized group*” OR “marginalized communit*” OR “underrepresented group*” OR “racial minorit*” OR immigrant* OR newcomer* OR refugee* OR “asylum seeker*” OR “displaced child*” OR “migrant child*” OR “forced migration”))** AND ((systematic OR scoping) AND review). The search was conducted from 2017 to May 31 2025 to reflect more recent discourse in terminology and methods for research with these communities. The search was restricted to studies published between Jan 1 2014 and May 31 2025.

Low- and Middle-Income Countries (LMICs): We searched for studies on interventions in LMICs using the following search terms: (autism OR "autism spectrum disorder" OR ASD OR asperger* OR PDD OR "pervasive developmental disorder") AND (diagnosis OR screening OR intervention OR therapy OR services) AND ("low and middle income countries" OR LMIC OR "low-income" OR "middle-income" OR Africa OR India OR Latin America OR Southeast Asia OR "global south") AND ("community engagement" OR "participatory action research" OR "community-based participatory research" OR "task-shifting" OR "parent-mediated"). The search was restricted to studies published between Jan 1 2014 and May 31 2025.
