# Supplementary material for: Advances in supporting development in autistic children and youth
Source: BMJ. 2026 Jun 10;393:e086562. doi: 10.1136/bmj-2025-086562 (PMC13250720; doi:10.1136/bmj-2025-086562)
Supplement: Supplementary file 2 — Appendix 2: Systematic reviews of intervention studies for preschool children [file penm086562.w2.pdf]

Appendix 2: Systematic reviews of Intervention Studies (Preschool) (2019-2025)

Reference	Focus	No. Papers	Total Subjects	Key Findings	Notes
Chetcuti et al. (2025) (1)	Design factors in intervention studies, and characteristics of families and children leading to outcomes of behavioural interventions for autistic children.	95 studies, 47 of which were RCTs	6,780 children age 7 to 87 mo	Post-intervention effects were higher in:<br>–Cognitive/language ability (QM =13.1, p=0.002<br>–Social skills (QM = 27.0, p < 0.001)<br>-Stronger post-intervention effects for longer duration (QM = 21.6, p < .001) and greater total hours in intervention (QM = 6.4, p < .001)	
Deniz et al. (2022) (2)	Effectiveness of play-based interventions on communication and language skills, and autistic traits of autistic preschool-aged children.	26 studies (21 in the meta-analysis), 23 of the studies were RCTs	1,459 children age 12 to 72 mo	Improvements in<br>–Social communication (d = 0.63, 95% CI [0.21, 1.05])<br>–Language skills (d = 0,40, 95% CI [0.09, 0.71])<br>–Autistic traits (d = -0.19, 95% CI [-0.34, -0.03])	
Franz et al., (2022) (3)	Early intervention for very young children with or at high likelihood for autism spectrum disorder: An overview of reviews	7 systematic reviews (published Jan 1, 2009 to Dec 31, 2020) including 63 studies	Children < 36 mo with or at high likelihood for ASD	Narrative synthesis, focused on heterogeneity in study design, intervention, and measurement. Only one of the 7 reviews included a meta-analysis, with improved development and behavior across various NDBI, developmental, behavioral, sensory, and technology-based intervention approaches (primary studies included single-participant designs; quality not evaluated)	Publication years did not overlap with search period for reviews reported in this paper (i.e. 2019, 2020)
Kulasinghe et al. (2023) (4)	Efficacy of psychological interventions to improve mental health of mothers of autistic children, and parent-child relationships.	32 RCTs<br><br>12 articles reported on mother's mental health, 13 articles on parent-child	2,336 children age 8.9 - 58.3 mo	-Reduced parent stress (d = -0.46 to -0.49, 16 studies) but no improvement in parental mental health<br>-Increased parental responsiveness (d = 0.49-1.10, 13 studies), increased parental affect (d = 0.47-1.17, 4 studies), reduced parental directiveness (d = 1.34 - -0.10, 4	Child outcomes independent of parent-child interaction not reported in this review

		relationships, and 7 articles on both<br><br>Included meta-analysis		studies) and increased child initiation (d=0.23 - 0.74, 8 studies), but not child affect or attentiveness	
Law et al. (2022) (5)	To determine the effectiveness of parent-mediated interventions for children with an increased likelihood of autism or with confirmed diagnoses	7 RCTs	457 children age 6 to 31 mo	Improvement in measures of parent interaction but not measures of infant autistic features nor rates of autism diagnosis. Variable effects on language and communication skills.	Not a meta-analysis; heterogeneity in effect sizes, measures of effect size and outcome measures among included studies made it difficult to draw conclusions
McGlade et al. (2023) (6)	Effectiveness of very early interventions for toddlers with an increased likelihood or a confirmed diagnosis of autism	19 papers from 12 studies, all were RCTs. Included meta-analysis	715 children age 6 - 23.9 mo	Clinician-assessed outcomes did not show significant effects:<br>–ADOS scores: MD: -0.08, 95% CI [-0.61, 0.44]<br>–Mullen Early Learning Composite: SMD: 0.05, 95% CI [-0.19, 0.29]<br><br>Improvement in parent reported adaptive skills:<br>- VABS-2: SMD=0.32, 95% CI (0.05-0.59)	
Ouyang et al. (2024) (7)	Effectiveness of parental-mediated naturalistic developmental behavioural interventions (NDBIs) in preschool autistic children	32 studies, all were RCTs, with meta-analysis	1,743 participants age 6-60 mo	Effect sizes for NDBI across intervention subtypes (compared to TAU)<br>- Language skills: SMD = 0.40, 95% CI (0.15, 0.65) - - - Social skills: SMD: 0.49, 95% CI (0.18, 0.80])<br>-Motor skills: SMD = 0.48, 95% CI (0.21, 0.74)	Subset of studies included active Parent Education comparison group; only effect detected was for Social Skills (SMD = 0.41, 95% CI [0.07 – 0.74]). High rates of parent fidelity reported for all intervention subtypes

Pak et al. (2023) (8)	Effectiveness of language and communication interventions in preschool-aged children both with autism (13 studies) or other language delays (7 studies) with outcome assessment at least 3 months later	20 studies, 14 of which were RCTs. Included meta-analysis.	1,639 children (999 were autistic) age 10.6 - 58.3 mo	Overall effect size: g: 0.22 (95% CI [0.10, 0.34] across intervention approaches	Overall prelinguistic outcomes were higher than linguistic outcomes (g: 0.36, 95% CI [0.19, 0.52] vs g: 0.14 95% CI [-0.03. 0.31])
Rants et al. (2024) (9)	Effectiveness of the Program for the Education and Enrichment of Relational Skills (PEERS) program in preschool autistic children (scoping review)	4 papers based on 5 studies, none were RCTs	82 children age 4 – 7 yr	The reviewed studies showed trends towards positive skill development, only one meeting statistical significance.	Effect sizes not reported for most studies.
Rodgers et al. (2021) (10)	Effectiveness of Applied Behavioural Analysis interventions (including NDBI) in autistic children (individual participant meta-analysis)	10 studies (of 15 eligible studies; 5 were excluded as individual participant data were not available). None were RCTs	491 participants mean age 38.4 mo	Increased adaptive skills (VABS) at 2 years (but not 1 year), increased IQ at 1 and 2 years, language effects varied, depending on measure reported	All ten studies were non-randomised and rated as being at 'serious' risk of bias for at least one domain
Zhao et al. 2025 (11)	Effectiveness of parent-mediated interventions (PMI; inclusive of NDBI, developmental and TEACCH models) and response moderators in autistic children <3 yr.	31 RCTs, 26 included in meta-analysis	1,468 parent–child pairs (721 who received active intervention), child age 7-36 mo.	Trivial-small positive effects (Hedges *g*) in several subdomains, including adaptive skills ($g = 0.29$), parent–child interaction ($g = 0.35$), social communication ($g = 0.18$), and symptoms ($g = -0.22$) but not children's cognitive competence, language, or motor skills. No intervention-related moderators reported.	Wide range of duration (2-108 weeks) and total hours (5-960) makes it difficult to compare studies.<br>Age range implies some children not yet diagnosed.
Zhou et al. (2024) (12)	Efficacy of social stories for preschool-aged children with or without an autism	21 studies (2 included in the meta-analysis – one	921 children age 2 – 6 yr	-Most studies reported improvements in targeted skills/behaviours	Included single subject design studies

	diagnosis (17 studies included autistic children); range of targeted skills (social, daily living including toothbrushing skills)	controlled trial, one RCT, both related to toothbrushing skills)		-The meta-analysis found improvement in oral hygiene (MD: 0.66, 95% CI [0.30, 1.02]).	

ADOS: Autism Diagnostic Observation Schedule; ADHD: attention-deficit/hyperactivity disorder; CI: confidence interval; CP: cerebral palsy; d: Cohen's d (effect size); g: Hedge's g (effect size); ID: intellectual disability; IQ: intelligence quotient; MD: mean difference; mo: months; NDBI: naturalistic developmental behavioural intervention; PMI: parent-mediated intervention; QM: test for moderators; RCT: randomized controlled trial; SMD: standardized mean difference; TAU: treatment as usual; VABS: Vineland Adaptive Behavior Scales; yr: years

References:

1. Chetcuti, L., Uljarević, M., Schuck, R. K., Hardan, A. Y., Gengoux, G. W., Trembath, D., Vadgama, Y., Varcin, K. J., Vivanti, G., Whitehouse, A. J. O., Helton, M., & Frazier, T. W. (2025). Characterizing predictors of response to behavioral interventions for children with autism spectrum disorder: A meta-analytic approach. Clinical psychology review, 119, 102588. https://doi.org/10.1016/j.cpr.2025.102588
2. Deniz E, Francis G, Torgerson C, Toseeb U. Parent-mediated play-based interventions to improve social communication and language skills of preschool autistic children: A systematic review and meta-analysis protocol. Didden R, editor. PLOS ONE. 2022 Aug 15;17(8):e0270153.
3. Franz L, Goodwin CD, Rieder A, Matheis M, Damiano DL. Early intervention for very young children with or at high likelihood for autism spectrum disorder: An overview of reviews. Dev Med Child Neurol. 2022 Sep;64(9):1063-1076. doi: 10.1111/dmcn.15258
4. Kulasinghe K, Whittingham K, Mitchell AE, Boyd RN. Psychological interventions targeting mental health and the mother–child relationship in autism: Systematic review and meta-analysis. Dev Med Child Neurol. 2023 Mar;65(3):329–45.
5. Law ML, Singh J, Mastroianni M, Santosh P. Parent-Mediated Interventions for Infants under 24 Months at Risk for Autism Spectrum Disorder: A Systematic Review of Randomized Controlled Trials. J Autism Dev Disord. 2022 Jun;52(6):2553–74
6. McGlade A, Whittingham K, Barfoot J, Taylor L, Boyd RN. Efficacy of very early interventions on neurodevelopmental outcomes for infants and toddlers at increased likelihood of or diagnosed with autism: A systematic review and meta-analysis. Autism Res. 2023 Jun;16(6):1145–60.
7. Ouyang Y, Feng J, Wang T, Xue Y, Mohamed ZA, Jia F. Comparison of the efficacy of parent-mediated NDBIs on developmental skills in children with ASD and fidelity in parents: a systematic review and network meta-analysis. BMC Pediatr. 2024 Apr 25;24(1):270.

8. Pak NS, Chow JC, Dillehay KM, Kaiser AP. Long-Term Effects of Early Communication Interventions: A Systematic Review and Meta-Analysis. J Speech Lang Hear Res. 2023 Aug 3;66(8):2884–99.
9. Rants S, Bradish K, Conlin H, Crandall N, Kirby N, Williams RM. PEERS® Curriculum for Children with Autism Spectrum Disorder: A Scoping Review. Phys Occup Ther Pediatr. 2024 Nov;44(6):865–73.
10. Rodgers M, Simmonds M, Marshall D, Hodgson R, Stewart LA, Rai D, Wright K, Ben-Itzchak E, Eikeseth S, Eldevik S, Kovshoff H, Magiati I, Osborne LA, Reed P, Vivanti G, Zachor D, Couteur AL. Intensive behavioural interventions based on applied behaviour analysis for young children with autism: An international collaborative individual participant data meta-analysis. Autism. 2021 May;25(4):1137-1153. doi: 10.1177/1362361320985680
11. Zhao X, Kuo F, Chen X, Li D, Duan H. Parent-Mediated Interventions for ASD Under 3 Years: A Systematic Review, Meta Analysis, and Moderator Analyses. J Autism Dev Disord. 2025 May 13. doi: 10.1007/s10803-025-06845-5
12. Zhou N, Zhou L, Ho CYT, McGrath C, Wong HM. Social Story Intervention for Training Expected Behaviors among Preschool Children: A Systematic Review and Meta-Analysis. Int J Environ Res Public Health. 2024 Jul 19;21(7):940.
