# Supplementary material for: Advances in supporting development in autistic children and youth
Source: BMJ. 2026 Jun 10;393:e086562. doi: 10.1136/bmj-2025-086562 (PMC13250720; doi:10.1136/bmj-2025-086562)
Supplement: Supplementary file 3 — Appendix 3: Systematic reviews of intervention studies for school-aged children and adolescents [file penm086562.w3.pdf]

Appendix 3: Systematic Reviews of Intervention Studies for School-Aged Children and Adolescents

Reference	Focus	No. Papers	Total Subjects	Key Findings	Notes
Alahmari et al. (2024) (1)	Social skills training programs for autistic children	17 RCTs and quasi-RCTs	Total 1269 participants (each study range 11 to 302)<br><br>age: 12 mo - 25 yr	Effect sizes ranged from 0.28-0.60; 95% CI 0.23-0.41, Meta-analysis focused on 4 studies that reported the SRS; not statistically significant.	Interventions and age ranges highly heterogeneous
Cavalli et al. (2022) (2)	Executive function intervention efficacy in performance in autistic children and adolescents	6 studies<br>3 RCTs	288 participants<br><br>Mean age by study 4 - 15.8 yr	Improvements in working memory, behaviour, and flexibility (reported descriptively)	Meta-analysis based on single study
Cheng et al. (2025) (3)	Program for the Education and Enrichment of Relational Skills (PEERS) on autistic adolescents and young adults, (looking at cultural and regional contexts)	21 RCTs and 10 non-RCTs	261 autistic adolescents and young adults<br><br>Mean age by study 12.7 - 27.6 years	Knowledge of social skills, application of social skills, and emotional intelligence. Smallest effects using PEERS were shown in East Asia<br>-Effect Sizes (Hedges' g):<br>–Overall TASSK effect size: 2.20 (95% CI [1.91, 2.49])<br>–Overall SRS effect size: -0.30 (95% CI [-0.43, -0.18])	Differences in teacher and caregiver responses were noted to be significant.
Cordier et al. (2023) (4)	Intervention studies that measured friendship outcomes for a variety of neurodevelopmental needs, including autism	12 RCT studies involving 15 interventions	683 children with a neurodevelopmental condition and 190 typically-developing children<br><br>age: 6 - 17 years	Individual interventions can increase social functioning and friendships between children with autism (*g*=0.485) and their peers (*g*=0.215)	

Darling et al. (2021) (5)	Behavioural interventions on children with neurodevelopmental or mental health conditions on social functioning and social cognition	33 studies, 31 of which were RCTs and 23 of which focused on autism.	1,743 participants<br><br>age: 4 to 17	Individuals in intervention groups tended to have improved social functioning and social cognition compared to control groups (Hedges' g effect size of 0.61, 95% CI [0.40, 0.83] and in the social function studies; 0.67 (95% CI [0.39, 0.96])	
Fan et al. (2023) (6)	Nature-based interventions that considered health outcomes (social, behavioural, emotional, and sensory functioning)	24 studies including 7 RCTs	717 participants<br><br>age: 5 - 17 yrs	Improvements in<br>–Social Communication: -0.59 (95% CI: [-0.85, -0.34])<br>–Hyperactivity: -0.56 (95% CI: [-0.86, -0.26])<br>–Irritability: -0.49 (95% CI [-0.79, -0.19])<br>–Inattention and Distractibility: 1.13 (95% CI: [0.67, 1.60])<br>–Sensory seeking: 0.77 (95% CI: [0.33, 1.22])<br>–Sensory sensitivity: 0.56 (95% CI: 0.12, 1.00])	
Geretsegger et al. (2022) (7)	Music Therapy<br>- duration: 3 days to 8 mo	26 RCTs and CCTs	1,165 participants<br>- 21 studies: 2-12 years; 5 studies included teens/adults	Positive global improvement (RR 1.22, 95% CI [1.06, 1.40]) with music therapy.<br><br>Reduction in autism features; no improvement in social interaction or verbal or non-verbal communication, slight increase in QoL	Outcomes assessed immediately after intervention (mean 3.4 mo)<br>- Serious methodological limitations: poor reporting and/or of randomisation in most studies
Gilmore et al. (2022) (8)	Impact of group social skills interventions for autistic youth and other developmental conditions	16 RCTs	1,119 participants	- Reduction of social impairment on the SRS (MD 9.68, 95% CI [5.63-13.73])<br>- Increased social skills on the SSIS (SMD 0.38, 95% CI [0.10-0.65]).	One study looked at a population of brain tumor survivors; the other fifteen looked at autistic youth.

Gosling et al. (2022) (9)	Impact of psychosocial interventions for autistic children and adolescents in an umbrella review looking at core and related features of autism.	128 meta-analyses from 44 reports	Not Applicable (review of meta-analyses)<br><br>Age: <6 - >20 yrs	Social skill groups tended to be significant in improving features such as social communication, IQ, and adaptive behaviours, with more significant improvements being shown in younger age groups.	Out of all the meta-analyses, only four had a statistically significant pooled effect size restricted to RCTs at low risk of detection bias.
James et al. (2020) (10)	Impact of cognitive behaviour therapy on children and youth with anxiety and with intersecting autism or intellectual disability	87 studies - 12 focused on autism	5,964 across all studies, not specified how many autistic children<br><br>age: 36 mo - 18 yr	Cognitive behaviour therapy is most effective when there is a short-to-no waitlist period and may be more effective than attention control. There is no evidence that cognitive behaviour therapy is *more* effective than other treatments	
Kouroupa et al. (2022) (11)	Robot-mediated interventions for children and adolescents. Examined robot platforms utilized, the role of robots, and range or outcomes	40 studies including 12 RCTs	854 children<br><br>age: 1.64 - 18 yrs	Humanoid robots in trials conducted in clinics provided the best measurable outcomes for the children in social functioning (Hedges g effect size: 0.35 (95% CI [0.09, 0.61]))	Small sample sizes (maximum participants per trial = 30)
Kou et al. (2024) (12)	Impact of physical activity interventions on autistic features	38 RCTs or quasi-RCTs	1,454 children and adolescents (740 in experimental groups, 714 in control groups)<br><br>age: 3 - 15 yr	Significant group differences in combination therapy [SMD=1.57, 95% CI (0.74, 2.40)], sports games [SMD=1.01, 95% CI (0.45, 1.56)], group ball games [SMD=0.85, 95% CI (0.45, 1.26)], outdoor exercise [SMD=0.79, 95% CI (0.48, 1.11)], and mind-body exercise [SMD=0.79, 95% CI (0.29, 1.30)]	

Liang et al. (2022) (13)	Impact of exercising interventions on the executive functioning of autistic children and youth	14 studies included in the systematic review, and 7 in meta-analysis	310 children<br><br>age: 6 - 18 yr	Chronic exercise interventions had a small to moderate effect on executive functioning of autistic children (cognitive flexibility and inhibitory control).<br>-For executive function: Hedges' g effect size: 0.342 (95% CI [0.084, 0.600])	
Liu et al. (2020) (14)	Impact of parent-mediated intervention to help parents gain strategies in supporting behavioral management and interaction in Asian countries	21 studies included in the systematic review, and 14 in the meta-analysis (16 were RCTs)	709 children and their caregivers; children aged 1 - 15 yrs	-Effect sizes (SMD)<br>–Symptom Severity: 1.00 (95% CI [0.77, 1.23])<br>–Language-communication: 0.64 (95% CI [0.48, 0.81])<br>–Social competence: 0.63 (95% CI [0.44, 0.83])<br>–Cognitive competence: 0.83 (95% CI [0.35, 1.31])<br>–Adaptive behaviour: 0.68 (95% CI [0.11, 0.81])	
McDaniel et al. (2022) (15)	Effectiveness of responsibility intervention techniques for improving prelinguistic and/or language outcomes in school-aged autistic children	67 studies - 33 RCTs and 34 single-case research design studies	897 unique participants<br><br>mean age 43.01 months (SD=17.97 months)	Improved pre-linguistic and language outcomes<br><br>Effect size g=0.36 (95% CI [0.21, 0.51])	Context-bound outcome measures had larger mean effect sizes than those with generalized outcome measures.
Mittal et al. (2024) (16)	Impact of virtual reality-based interventions on autistic children and adolescents in cognitive, social, and emotional domains	6 RCTs	422 participants<br><br>age: 2 - 17 years	Improved cognitive, social, and emotional skills of autistic children<br><br>SMD for emotional skills: 2.20 (95% CI ([0.21, 4.18])<br>SMD for social skills: 1.43 (95% CI [0.01, 2.84])	

Nuske et al. (2024) (17)	Interventions that target emotional dysregulation and challenging behaviours	96 studies, including 29 group and 66 single case designs - 15 were RCTs	2,092 participants across four continents with an autism diagnosis, age: 3 – 18 yr	Parent-implemented intervention, emotional regulation training, reinforcement, visual supports, cognitive behavioural/instructional strategies, and antecedent-based interventions were shown to be the most effective interventions in improving challenging behaviour outcomes and emotional regulation outcomes	Outcome measures are not comparable across the studies
Pi et al. (2022) (18)	Parent mediated technology assisted interventions in improving social communication and interaction outcomes in school-aged autistic children	16 RCTs	748 participants<br><br>age 18 mo - 23 yrs	-No statistically significant differences in social communication (RR 0.75 [-0.16, 1.66]), social functioning (RR 1.83 [-2.01, 5.68]), or language outcomes (MD -0.06 (95% CI [-2.76, 2.64]).	
Pruneti et al. (2024) (19)	The impact of behavioural therapy interventions in autistic children in many different domains	17 including 3 RCT controlled (intervention vs traditional), 3 RCT comparison, comparing several types of interventions	768 participants<br><br>age 11 mo - 18 yr	Some evidence for Picture Exchange Communication System and Pivotal Response Treatment to improve receptive language, adaptive behavior, daily living skills, verbal and non-verbal intelligence quotient, restricted and repetitive behavior, and motor and cognition,	
Rehn et al. (2023) (20)	Impact of animal-assisted therapy on school-age autistic children's cognitive, social, emotional,	7 RCTs	340 participants (age ranges 2 - 16 yrs)	There were overall positive shifts in all domains: cognitive, social, emotional, behavioural, and physical. Inconsistent reporting on effect sizes, with two studies noting small-to-medium effect sizes.	Due to the heterogeneity of intervention protocols and outcomes measured, meta-analysis was not possible

	behavioral, and physical domains.				
Rosales et al. (2025) (21)	Impact of motor intervention on social, communication, and cognitive skills in autistic individuals	23 RCTs (15 included in meta-analysis)	636 participants<br><br>Mean ages by study 4.3 to 12.3 yr	Positive effect on:<br>- Social measures (SSMD: 0.46, p = .012)<br>- Social/communication measures (SSMD: 0.47, p = .01)<br>- No effect on motor (SSMD: 0.45, p = 0.25) or cognitive measures (SSMD: 0.22, p = 0.18).	Greater intervention effects in children <9 years vs older
Sharma et al. (2021) (22)	Cognitive behaviour therapy specifically looking at co-occurring anxiety in autistic youth	19 RCT studies	833 participants<br><br>Mean ages from 5.42 - 15.56 yrs	Reduced anxiety in the immediate intervention period: Effect sizes (Hedges' g):<br>–Parent rated measures of anxiety: 0.40 (95% CI [0.24, 0.56])<br>–Clinician rated measures of anxiety: 0.88 (95% CI [0.55, 1.21])<br>–Child rated measures of anxiety: 0.25 (95% CI [0.06, 0.43])	
Sissons et al. (2022) (23)	Impact of animal-based interventions on school age autistic children's social functioning.	9 RCTs across 8 trials	516 participants<br><br>age: 4 - 18 years	Gains in some domains of social functioning (SRS and Vineland Adaptive Behavior Scales subscales)<br>-Effect size calculated as (2 × t-value)/√df from the contrast of the time × group interaction = 0.421 (statistically significant)	Gains were maintained in the follow-up studies that were reported
Tao et al. (2025) (24)	Impact of various physical activity interventions on children with various neurodevelopmental disorders and conditions, including autism on cognitive abilities	31 RCTs	1,403 children with neurodevelopmental conditions including autism and ADHD<br><br>Mean age 10.0 ± 1.9 yr	-Mind-body exercise SMD = 1.91 for attention, 0.92 executive functions<br>-Exergaming SMD = 1.58 for attention, 0.94 for executive functions, 0.97 for memory<br>-Multi-component physical activity SMD = 0.79 for executive functions	

Tseng et al. (2020) (25)	Impact of social cognitive interventions on autistic adolescents (12-18 years old)	18 RCTs	Not specified	Social Skills effect sizes for intervention (PEERS in multiple languages/cultures and other group models) ranged from d=0.69-1.48 (moderate-large); no meta-analyses reported	
Wang et al. (2025) (26)	Impact of exercise therapy on executive functioning for autistic children and adolescents	16 RCTs	678 children and adolescents; 372 in experimental group<br><br>age 3 – 17 yr	Improvement in executive functioning: SMD = 0.41, 95% CI (0.30, 0.52)	No differences in subgroup analysis for: medication usage, exercise characteristics
Wang et al. (2021) (27)	CBT: effectiveness on autism symptoms and social-emotional problems	45 RCTs and 6 quasi RCTs	2,485<br><br>age range not specified	No difference between CBT and control for autism features based on self-report (SMD: 20.09; 95% CI: 20.42 to 0.24; p= 0.59)<br><br>CBT improved autism symptoms and social-emotional symptoms (informant- and clinician-reported outcomes)	
Yang & Li (2025) (28)	Effectiveness of physical activity interventions on repetitive behaviours in autistic children	20 RCTs	671 autistic children<br><br>Mean ages 1.0 to 12.4 years	Exercise intervention had a positive effect on repetitive stereotyped behaviors in autistic patients (SMD = −0.37, 95% CI: −0.52, −0.21).<br>-Effect positively moderated by ball sports (SMD = −0.72), longer duration (SMD = −0.55), higher frequency (SMD = −0.74), longer time (SMD = −0.84), and group participation (SMD = −0.48)	Heterogeneity in participant age, intervention type, outcome measures and study quality (e.g., not all applied intent to treat in analyses)

CBT: cognitive behavioural therapy; CCTs: quasi-randomised controlled clinical trials; CI: confidence interval; g: Hedge's g (effect size); IQ: intelligence quotient; MD: mean difference; mo: months; PEERS: Program for the Education and Enrichment of Relational Skills; QoL: quality of life; RCT: randomised controlled trial; RR: relative risk: SMD: standardized mean difference; SRS: Social Responsiveness Scale; SSIS: Social Skills Improvement System; SSMD: strictly standardised mean difference: TASSK: Test of Adolescent Social Skills Knowledge; VR: virtual reality; yr: years

References

1. Alahmari FS, Alhabbad AA, Alshamrani HA, Almuqbil MA. Effectiveness of social skills training interventions for children with autism spectrum disorder: A systematic review and meta-analysis. Saudi Med J. 2025 Mar;46(3):226–37
2. Cavalli G, Galeoto G, Sogos C, Berardi A, Tofani M. The efficacy of executive function interventions in children with autism spectrum disorder: a systematic review and meta-analysis. Expert Rev Neurother. 2022 Jan 2;22(1):77–84.
3. Cheng Y, Shi J, Cheng X, Wei Y, Wang J, Jiang Z. Impact of social knowledge and skills training based on UCLA PEERS® on social communication and interaction skills of adolescents or young adults with autism: A systematic review and meta-analysis. Asian J Psychiatry. 2025 Apr;106:104422.
4. Cordier R, Parsons L, Wilkes-Gillan S, Cook M, McCloskey-Martinez M, Graham P, et al. Friendship interventions for children with neurodevelopmental needs: A systematic review and meta-analysis. Huerta-Quintanilla R, editor. PLOS ONE. 2023 Dec 14;18(12):e0295917.
5. Darling SJ, Goods M, Ryan NP, Chisholm AK, Haebich K, Payne JM. Behavioral Intervention for Social Challenges in Children and Adolescents: A Systematic Review and Meta-analysis. JAMA Pediatr. 2021 Dec 6;175(12):e213982.
6. Fan MSN, Li WHC, Ho LLK, Phiri L, Choi KC. Nature-Based Interventions for Autistic Children: A Systematic Review and Meta-Analysis. JAMA Netw Open. 2023 Dec 7;6(12):e2346715.
7. Geretsegger M, Fusar-Poli L, Elefant C, Mössler KA, Vitale G, Gold C. Music therapy for autistic people. Cochrane Developmental, Psychosocial and Learning Problems Group, editor. Cochrane Database Syst Rev [Internet]. 2022 May 9 [cited 2025 Jun 26];2022(5). Available from: http://doi.wiley.com/10.1002/14651858.CD004381.pub4
8. Gilmore R, Ziviani J, Chatfield MD, Goodman S, Sakzewski L. Social skills group training in adolescents with disabilities: A systematic review. Res Dev Disabil. 2022 Jun;125:104218.
9. Gosling CJ, Cartigny A, Mellier BC, Solanes A, Radua J, Delorme R. Correction: Efficacy of psychosocial interventions for Autism spectrum disorder: an umbrella review. Mol Psychiatry. 2022 Sep;27(9):3657–3657.
10. James AC, Reardon T, Soler A, James G, Creswell C. Cognitive behavioural therapy for anxiety disorders in children and adolescents. Cochrane Common Mental Disorders Group, editor. Cochrane Database Syst Rev [Internet]. 2020 Nov 16 [cited 2025 Jun 25];2020(11). Available from: http://doi.wiley.com/10.1002/14651858.CD013162.pub2
11. Kouroupa A, Laws KR, Irvine K, Mengoni SE, Baird A, Sharma S. The use of social robots with children and young people on the autism spectrum: A systematic review and meta-analysis. Vassalle C, editor. PLOS ONE. 2022 Jun 22;17(6):e0269800.
12. Kou R, Li Z, Li M, Zhou R, Zhu F, Ruan W, et al. Comparative effectiveness of physical exercise interventions on sociability and communication in children and adolescents with autism: a systematic review and network meta-analysis. BMC Psychol. 2024 Nov 30;12(1):712.
13. Liang X, Li R, Wong SHS, Sum RKW, Wang P, Yang B, et al. The Effects of Exercise Interventions on Executive Functions in Children and Adolescents with Autism Spectrum Disorder: A Systematic Review and Meta-analysis. Sports Med. 2022 Jan;52(1):75–88.

14. Liu Q, Hsieh WY, Chen G. A systematic review and meta-analysis of parent-mediated intervention for children and adolescents with autism spectrum disorder in mainland China, Hong Kong, and Taiwan. Autism. 2020 Nov;24(8):1960–79.
15. McDaniel J, Brady NC, Warren SF. Effectiveness of Responsivity Intervention Strategies on Prelinguistic and Language Outcomes for Children with Autism Spectrum Disorder: A Systematic Review and Meta-Analysis of Group and Single Case Studies. J Autism Dev Disord. 2022 Nov;52(11):4783–816.
16. Mittal P, Bhadania M, Tondak N, Ajmera P, Yadav S, Kukreti A, et al. Effect of immersive virtual reality-based training on cognitive, social, and emotional skills in children and adolescents with autism spectrum disorder: A meta-analysis of randomized controlled trials. Res Dev Disabil. 2024 Aug;151:104771.
17. Nuske HJ, Young AV, Khan FY, Palermo EH, Ajanaku B, Pellecchia M, et al. Systematic review: emotion dysregulation and challenging behavior interventions for children and adolescents on the autism spectrum with graded key evidence-based strategy recommendations. Eur Child Adolesc Psychiatry. 2024 Jun;33(6):1963–76.
18. Pi HJ, Kallapiran K, Munivenkatappa S, Kandasamy P, Kirubakaran R, Russell P, et al. Meta-Analysis of RCTs of Technology-Assisted Parent-Mediated Interventions for Children with ASD. J Autism Dev Disord. 2022 Aug;52(8):3325–43.
19. Pruneti C, Coscioni G, Guidotti S. Evaluation of the effectiveness of behavioral interventions for autism spectrum disorders: A systematic review of randomized controlled trials and quasi-experimental studies. Clin Child Psychol Psychiatry. 2024 Jan;29(1):213–31.
20. Rehn AK, Caruso VR, Kumar S. The effectiveness of animal-assisted therapy for children and adolescents with autism spectrum disorder: A systematic review. Complement Ther Clin Pract. 2023 Feb;50:101719.
21. Rosales MR, Butera CD, Wilson RB, Zhou J, Maus E, Zhao H, Chow JC, Dao A, Freeman J, Dusing SC. Systematic Review and Meta-Analysis of the Effect of Motor Intervention on Cognition, Communication, and Social Interaction in Children with Autism Spectrum Disorder. Phys Occup Ther Pediatr. 2025;45(5):688-710. doi: 10.1080/01942638.2025.2498357
22. Sharma S, Hucker A, Matthews T, Grohmann D, Laws KR. Cognitive behavioural therapy for anxiety in children and young people on the autism spectrum: a systematic review and meta-analysis. BMC Psychol. 2021 Dec;9(1):151.
23. Sissons JH, Blakemore E, Shafi H, Skotny N, Lloyd DM. Calm with horses? A systematic review of animal-assisted interventions for improving social functioning in children with autism. Autism. 2022 Aug;26(6):1320–40.
24. Tao R, Yang Y, Wilson M, Chang JR, Liu C, Sit CHP. Comparative effectiveness of physical activity interventions on cognitive functions in children and adolescents with Neurodevelopmental Disorders: a systematic review and network meta-analysis of randomized controlled trials. Int J Behav Nutr Phys Act. 2025 Jan 13;22(1):6.
25. Tseng A, Biagianti B, Francis SM, Conelea CA, Jacob S. Social Cognitive Interventions for Adolescents with Autism Spectrum Disorders: A Systematic Review. J Affect Disord. 2020 Sep;274:199–204.
26. Wang H, Cheng G, Li M meng. The effectiveness and sustained effects of exercise therapy to improve executive function in children and adolescents with autism: a systematic review and meta-analysis. Eur J Pediatr. 2025 Apr 8;184(5):286.

27. Wang X, Zhao J, Huang S, Chen S, Zhou T, Li Q, et al. Cognitive Behavioral Therapy for Autism Spectrum Disorders: A Systematic Review. Pediatrics. 2021 May 1;147(5):e2020049880.
28. Yang J, Li R. Systematic review and randomized controlled trial meta-analysis of the effects of physical activity interventions and their components on repetitive stereotyped behaviors in patients with autism spectrum disorder. Front Psychol. 2025 May 23;16:1579345. doi: 10.3389/fpsyg.2025.1579345
